# Small supernumerary marker chromosomes derived from chromosome 14 and/or 22

**DOI:** 10.1186/s13039-021-00533-6

**Published:** 2021-02-25

**Authors:** Thomas Liehr, Heather E. Williams, Monika Ziegler, Stefanie Kankel, Niklas Padutsch, Ahmed Al-Rikabi

**Affiliations:** 1 Institute of Human Genetics, Jena University Hospital, Friedrich Schiller University, Am Klinikum 1, 07747 Jena, Germany; 2 Department of Pathology and Cell Biology, Columbia University Irving Medical Center, 622 West 168th Street, New York, NY 10032 USA

**Keywords:** Small supernumerary marker chromosomes (sSMCs), Chromosome 14, Chromosome 22, Prenatal, Postnatal, Incidence

## Abstract

Small supernumerary marker chromosomes (sSMCs) are additional derivative chromosomes present in an otherwise numerically and structurally normal karyotype. They may derive from each of the 24 human chromosomes, and most contain a normal centromeric region with an alphoid sequence from a single chromosome. The majority of human chromosomes have a unique centromeric DNA-sequence enabling their indubitable characterization. However, chromosomes 14 and 22 share a common centromeric sequence D14/22Z1, and sSMCs with this DNA-stretch can derive from either chromosome. Euchromatin-carrying sSMCs(14 or 22) may be further characterized by molecular cytogenetics. However, in most diagnostic laboratories, heterochromatic sSMCs cannot be differentiated between chromosomes 14 or 22 derivation and are often reported as der(14 or 22). Still, heterochromatic sSMC(14 or 22) can be distinguished from each other using the D22Z4 probe (non-commercial) localized to 22p11.2. Herein, 355 sSMC(14 or 22) analyzed in the authors’ laboratory during the last ~ 20 years are summarized to address the questions: (1) What are the true frequencies of chromosome 14- and chromosome 22- derived sSMCs within D14/22Z1-positive cases? (2) Does sub-characterization of sSMC(14) and sSMC(22) make a difference in routine diagnostics? These questions could be answered as follows: (ad 1) within the studied group of sSMCs ~ 40% are derived from chromosome 14 and ~ 60% from chromosome 22; (ad 2) the knowledge on exact sSMC origin can help to save costs in routine diagnostics; i.e. in a clinically abnormal person with sSMC(14) a test for uniparental disomy is indicated, which is not necessary if a chromosome 22 origin for the sSMC was determined.

## Background

Small supernumerary marker chromosomes (sSMCs) represent a rare aberration as they are simultaneously a numerical and structural rearrangement. sSMCs are additional derivative chromosomes present in an (in most cases) otherwise numerically and structurally normal karyotype. They can have a variety of sizes and structures derived from all 24 human chromosomes. Most carry one or two normal centromeric regions with alphoid sequences [1]. Although recognized since 1961 [2], sSMCs remain an issue in diagnostics. However, in the preceding decade, progress has been made regarding genotype–phenotype correlations for sSMC subgroups [1, 3]; sSMC related clinical syndromes have been identified e.g. cat eye syndrome (OMIM 115470), Emanuel syndrome (OMIM 609029), Pallister-Killian syndrome (OMIM 601803), isochromosome 18p-syndrome (OMIM 614290), supernumerary der(22)t(8;22)-syndrome (OMIM 613700) [4], and others [5]. The clinical outcome of the remaining sSMC cases is largely attributed to euchromatic content of the aberration. In particular, phenotypes have been associated with the presence (or absence) of dosage sensitive genes within pericentromeric euchromatic regions. Nonetheless, specific gene(s) have not yet been identified [6]. Furthermore, the occurrence of de novo sSMC in diagnostics is complicated by the following problems:

- sister chromosomes of a sSMC may be subject to uniparental disomy (UPD) [7];
- sSMC may by discontinuous, due to formation by chromothripsis; this makes sSMC cases unique, private events and genotype–phenotype correlations difficult, or nearly impossible [8];
- sSMCs may be complex, i.e. consist of a centromeric part of chromosome A and a telomeric part of chromosome B [5];
- sSMC-presence may hint towards a cryptic mosaic in certain body tissues; indeed tissues may harbor cells comprised of complete trisomy of the chromosome from which the sSMC originated (incomplete trisomic rescue) [9].

When an sSMC is detected via banding cytogenetics, nowadays there is often discussion of how to further characterize it. Molecular karyotyping seems at first glance to be optimal, as (if a SNP-based array is used) even isoUPD of an sSMC’s sister chromosomes can be detected, along with copy number gains induced by the sSMC [10]. In addition, discontinuous sSMCs can also be readily detected, along with cryptic mosaic forms of full trisomies. However, there are limitations to this approach as euchromatic sections of an sSMC (and potentially a mosaic trisomy) can only be detected if the percentage of cells containing the aberration is large enough to be detected by the corresponding applied platform. As mosaicism is rather rule than exception in sSMCs [11], which can impact phenotype [12], the authors’ laboratory remains committed to sSMC characterization by molecular cytogenetics. This holds true for the majority of cytogenetic institutions around the world, which given financial constraints often cannot afford modern and expensive newer high throughput approaches [13, 14].

While molecular cytogenetics has clear benefits, such as enabling single cell level studies, and thus has the potential to detect even low level and cryptic mosaics, there are also limitations [13, 14]. For example, sSMCs, clearly or not clearly derived from an acrocentric chromosome (based on silver staining of nucleolus organizer region [15]) cannot be unambiguously resolved for chromosomal origin via fluorescence in situ hybridization (FISH) with centromeric probes. While the chromosome 15 probes D15Z1 in 15p11.2 and D15Z3 in 15q11.1 guarantee clear results if the sSMC is derived from chromosome 15, this resolution is not possible between chromosomes 13 and 21 or 14 and 22, as they have common alphoid sequences, i.e. D13/21Z1 and D14/22Z1. Thus, the chromosome of origin cannot be determined between these chromosomes when the sSMC contains no euchromatin. While this problem is as of yet unresolvable between chromosomes 13 and 21, chromosomes 14 and 22 can be distinguished using the D22Z4 probe localized on 22p11.2, which is not commercially available [16]. Also it has to be added that molecular cytogenetics must be the gold-standard for sSMC-characterization, as molecular karyotyping may miss up to > 80% of sSMC, as recently shown [17].

Herein, 355 sSMC cases positive for D14/22Z1 via FISH analyzed in the authors’ laboratory during the last ~ 20 years (Additional file 2: Tables S1–S3—and sSMC database), were revisited. The main questions are: What are the true frequencies of chromosome 14- and chromosome 22–derived sSMCs among D14/22Z1-positive cases? In addition, we investigated whether sub-characterization of sSMC(14) and sSMC(22) makes a difference in routine diagnostics and counselling.

## Results

Overall, 355 sSMC derived from chromosomes 14 or 22 were studied, which comprised 172 clinically normal cases, 110 clinically abnormal, and 73 with unclear clinical correlation. Comparatively, prenatal detection included 45/110 cases (~ 41%) designated clinically abnormal, whereas although postnatal detection included a higher number of cases, there was a slightly lower proportion of clinically affected cases (65/185, ~ 35%). Please note that individuals with ‘infertility’ were considered clinically normal.

**Fig. 1:**
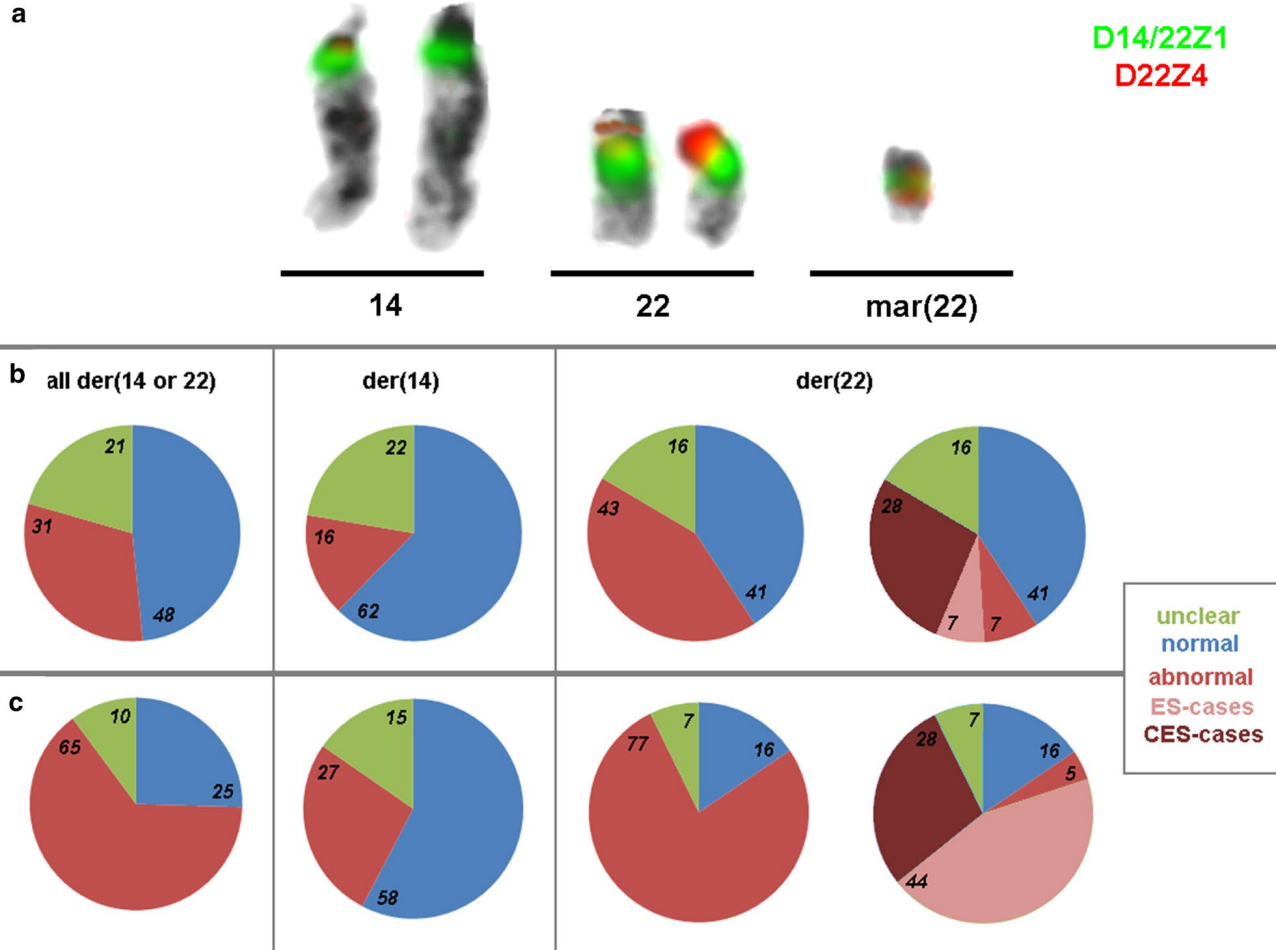
**a** A typical result after co-hybridization of D14/22Z1 (green), specific for 14p11.1-q11.1 and 22p11.1-q11.1 and D22Z4 (red), staining 22p11.2. On green signal is visible each on both chromosomes 14 and 22, as well as the sSMC (mar(22)); however, red signals are only present on both chromosomes 22 (one time, each), and two red signals on the sSMC. **b**, **c** Frequencies of sSMC cases characterized in this study (**a**) and in the literature (Liehr, 2020a) (**b**) are depicted. Percentages for cases with normal, abnormal, and unclear clinical outcomes are given for der(14 or 22), der(14) and der(22) carriers. For der(22) cases, the abnormal cases are on the left side and subdivided in cat eye syndrome (CES) and Emanuel syndrome (ES). In all pie charts the number of abnormal cases is higher in the literature (**c**) than cases from the authors’ single laboratory (**b**)

The D22Z4 probe localized to 22p11.2 could only aid in the distinction between an sSMC(14) or sSMC(22) if it contained 22p11.2 material; if this was not the case and/or insufficient material was available for further sSMC-characterization, the sSMC could not be clearly designated as a der(14) or der(22). An example of a case where the sSMC could be clearly characterized as a inv dup(22)(q11.1) after FISH is shown in Fig. 1A. This ambiguity occurred in 19/355 cases (5.4%) (Additional file 2: Table S1a-c). A normal outcome was documented in seven of nine cases, whereas in the ten remaining cases no clinical information was available.

sSMCs that lacked detectable signals for the D22Z4 probe, but had D14/22Z1 signals were considered derivatives of chromosome 14. For 101/130 cases clinical correlation was available: 81 cases had a normal outcome, while 20 demonstrated diverse cytogenomic aberrations and clinical outcomes. In 3/20 cases, the sSMC was present in conjunction with UPD(14), and in 8/20 cases, complex sSMCs were present. Interestingly, there were no statistically differences in the percentage of prenatally and postnatally detected abnormal versus normal sSMC carriers.

Heterochromatic sSMCs with detectable D22Z4 and D14/22Z1 FISH-signals were considered derived from chromosome 22. Euchromatic sSMCs anyway were positive for probes derived from 22q11.2 and thus clearly attributed to be derived from #22. While 34/206 cases lacked clinical information, the remaining 172 cases could be placed into several groups. As previously stated, there were two distinct groups composed of 84 clinically normal and 88 clinically abnormal carriers. Abnormal cases accounted for more prenatal (~ 58%) compared to postnatal cases (~ 47%). Within the abnormal cases there were two well defined syndromes: cat eye syndrome ((CES)—56 cases) and Emanuel syndrome ((ES)—15 cases). CES was detected prenatally in ~ 68% and postnatally in ~ 60% of the abnormal cases, and ES was detected prenatally in 22% and postnatally in 14%. In addition, there were 3 cases of prenatally detected sSMC carriers with complex sSMC(22). Interestingly, one clinically normal carrier of an sSMC derived from chromosome 22 also had UPD(22).

**Fig. 2:**
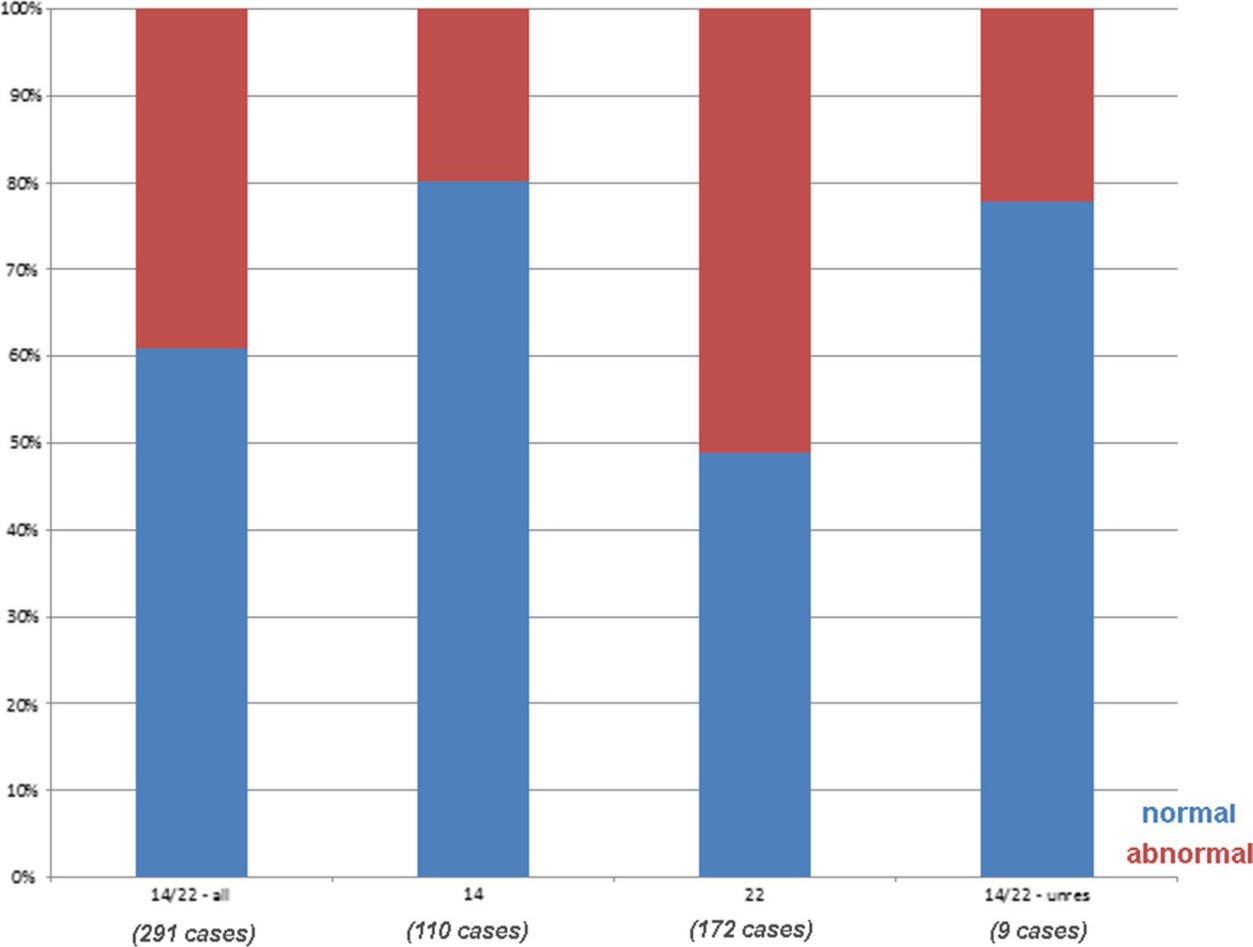
The 291/355 cases with clear clinical result studied herein are subdivided by normal and abnormal phenotypes. In first column all 291 cases are listed (14/22—all), the second and third columns include only sSMCs derived from chromosomes 14 or 22, i.e. der(14) or der(22), respectively; the last column includes unresolvable cases (14/22—unres). Normal cases are highlighted in blue, abnormal in red

**Fig. 3:**
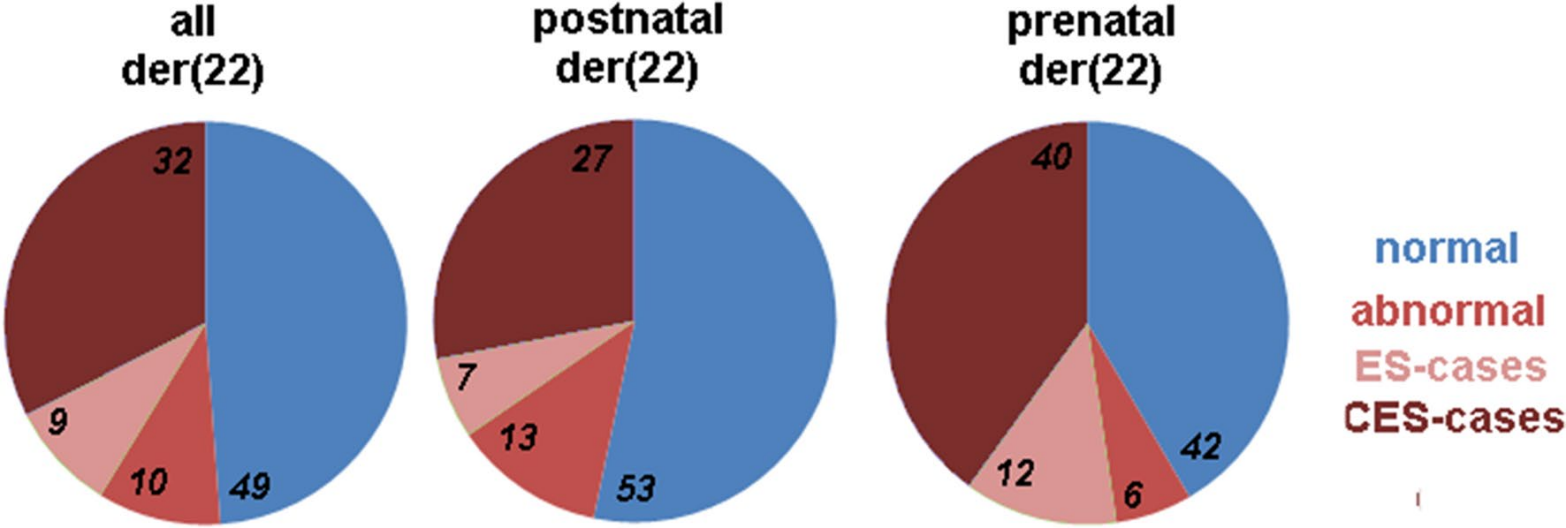
These pie charts depict firstly all der(22) cases with clear clinical correlation, which are subsequently compared to the relative frequencies of prenatal and postnatal detection

**Fig. 4:**
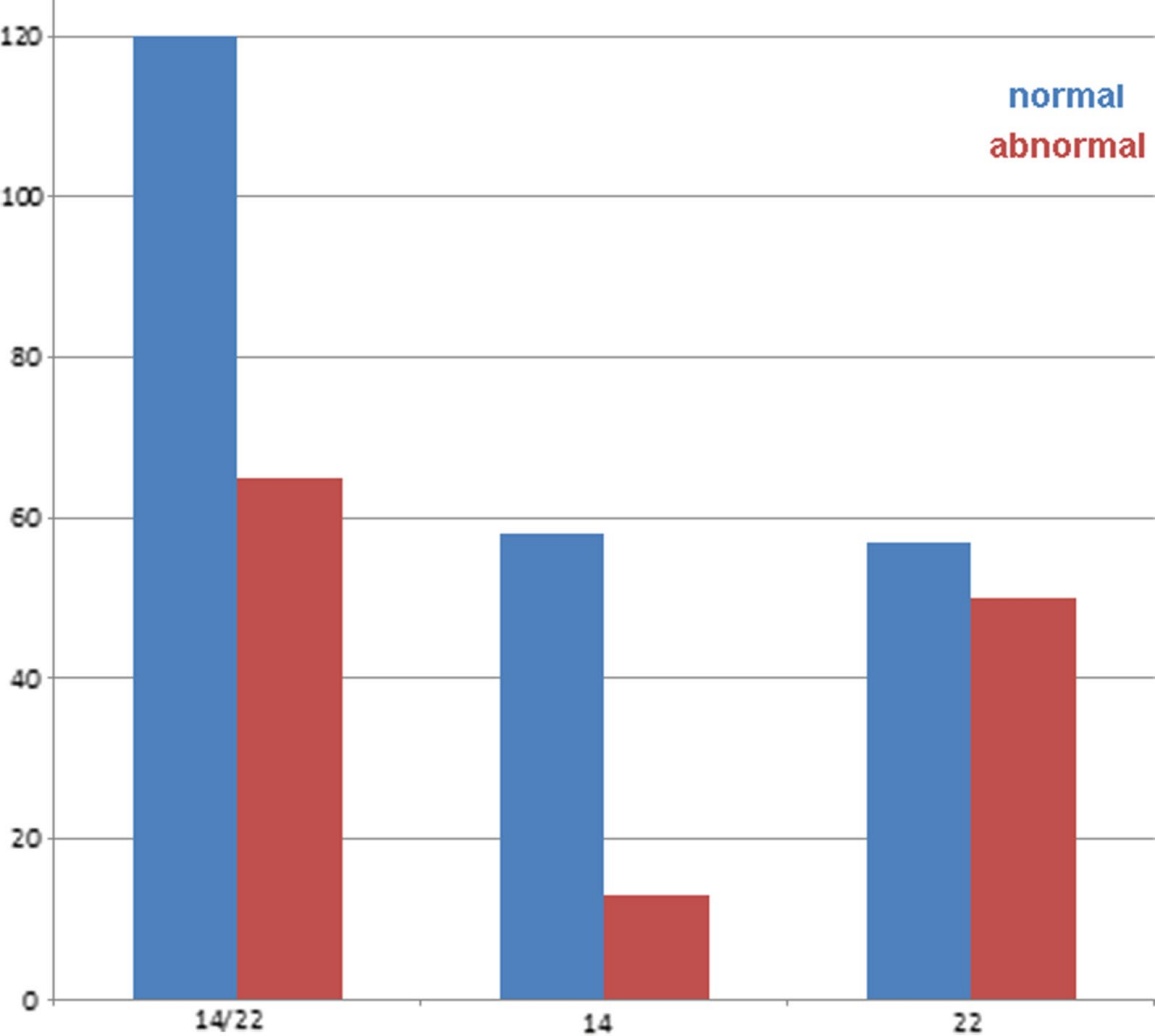
Normal and abnormal cases before (first double column) and after (second and third double columns) separation into either chromosome 14 or chromosome 22 derived sSMCs, and their correlation with normal and abnormal clinical outcome

Figure 1b depicts the distribution of clinically normal and clinically abnormal cases for all der(14 or 22), in combination with cases with unclear clinical correlation, including those sSMC characterized as der(14) or der(22). Figure 1c summarizes all published cases [3] for comparison, highlighting publication bias. Figure 2 summarizes the 291 cases with known clinical correlation of the sSMC. Figure 3 summarizes cases with chromosome 22 derived sSMCs, with differences in pre- and postnatal detection highlighted. Finally, Fig. 4 compares frequencies of normal and abnormal sSMC carriers with sSMC(14 or 22), sSMC(14) and sSMC(22).

## Discussion

Here the two questions raised at the beginning of this publication are answered.

**What are the true frequencies of chromosome 14- and chromosome 22-derived sSMCs within D14/22Z1-positive cases?**

Herein, 355 sSMC cases derived from chromosomes 14 or 22 from a single laboratory are summarized (Fig. 1b). This collective of patients has the advantage that it is not influenced by ‘publication-bias’, as otherwise present in the literature (Fig. 1c). The latter bias is a consequence of the report of only ‘interesting’ cases in scientific papers. Thus, it is more likely that clinically abnormal cases are reported than clinically normal ones; and such cases lost during follow-up they are not reported at all. Consequently, the data presented here should be a true representation of findings expected in a cytogenetic lab during routine diagnostics.

Nearly 40% of sSMC(14 or 22) cases detected in our laboratory were clinically abnormal (Fig. 2). Complete characterization of the sSMC as either sSMC(14) or sSMC(22) further divided the overall proportion of abnormal cases, as only 20% of der(14) compared with 52% of der(22) were clinically abnormal. Distinction between detection period (prenatal or postnatal) for der(14) versus der(22) was associated with only slight differences, which included the prenatal detection of 24% normal vs. 58% abnormal cases (Additional file 1: Fig. S1). Thus, there is a remarkable difference in risk estimation when the lab can make a determination that the sSMC of the fetus is derived from a chromosome 14 or 22 (with the risk for an affected child at 45%), versus those derived from either chromosome 14 (24%) or 22 (58%). It is estimated that 30–50% of fetuses with de novo sSMCs without clear clinical prognoses are terminated, and the assigning of the sSMC to the respective derivative chromosome is critical for pregnancy decision-making [18, 19]. Overall, within the here studied group of sSMC(14 or 22) carriers ~ 40% of sSMCs are derived from chromosome 14 and ~ 60% from chromosome 22 (Fig. 2).

**Does sub characterization of sSMC(14) and sSMC(22) make a difference in routine diagnostics?**

For routine diagnostics it must be considered that de novo sSMCs may also occur in conjunction with a UPD of sister chromosomes the sSMC derived from [7]. Exclusion of UPD is clinically paramount if the sSMC is derived from chromosome 14, as UPD(14)mat results in Temple syndrome (OMIM 616222), and UPD(14)pat in Kagami-Ogata syndrome (OMIM 608149) [4]. For chromosome 22, given the absence of imprinting defects, it is only the extremely rare homozygote recessive condition that results from (segmental) iso-UPD, which could have a clinical impact. However, only 11 of such cases are known, and none contained an sSMC [20]. Accordingly, a definite characterization of de novo sSMC(14) origin in a prenatal case or in a clinically abnormal person saves costs in routine diagnostics, as the number of patient-probes to be submitted to a UPD-test can markedly be reduced.

Furthermore, if an sSMC has been characterized to derive from chromosome 22 using centromeric probes without knowledge of the clinical presentation, it is more likely that in a postnatal setting a normal sSMC carrier (53%) has been detected, than when such an aberration has been detected in prenatal setting (42%); see Fig. 3. Interestingly, during the postnatal detection of clinically normal sSMC(14 or 22) carriers, when the sSMC is further characterized by the chromosome of origin, there is an approximate 1:1 ratio into mostly heterochromatic derivative chromosomes der(14) or der(22). Thus, during postnatal detection in clinically normal patients studied due to infertility, the clinical impact of detailed sSMC characterization is negligible and can be omitted.

Finally, it must be considered that the characterization of an sSMC being definitely derived from chromosome 14 or 22 is, in a prenatal setting and/ or a definitely clinically abnormal sSMC-carrier only the starting point to further characterize the genetic content of an sSMC. However, it is always possible that a heterochromatic sSMC, where also a possibly meaningful UPD has been excluded, may be only an anecdotal finding, as the real reason for the observed clinical problems of the patient have another genetic reason, like previously reported by us for an sSMC-carrier with fragile-X-syndrome [21].

## Conclusion

In conclusion, further characterization of a prenatally detected sSMC(14 or 22) by the heterochromatin-directed D22Z4 probe and/or euchromatin directed probes in the long arm of chromosome 14 or 22 is of the utmost importance; these studies provide essential information on the putative clinical outcome of the unborn child and should be performed. Finally, only cases with the sSMC derived from chromosome 14 require testing to exclude UPD(14).

## Material and methods

This retrospective study included 355 sSMC cases. All cases were positive for D14/22Z1 via FISH and were analyzed in the authors’ laboratory during the last ~ 20 years (Additional file 2: Tables S1–S3). All were previously included in the sSMC database [3] and/or other literature referred to in Additional file 2: Tables S1–S3. Nevertheless, all sSMC were further characterized with the D22Z4 probe, specific for 22p11.2, and/or, if euchromatic, by other probes as listed in sSMC database [3].

The Additional file 2: Table S1a-c include sSMCs derived from chromosomes 14 or 22, Additional file 2: Table 2a-c those derived from chromosome 14, and Additional file 2: Table S3a-c those derived from chromosome 22. Additional file 2: Table S1a, S2a and S3a include cases which were clinically normal, Additional file 2: Table S1b, S2b and S3b include clinically abnormal cases (including those with cat eye syndrome and Emanuel syndrome), and Additional file 2: Tables S1c, S2c and S3c include cases which have not been associated with a clear clinical outcome, due to loss during follow-up.

## Supplementary Information

**Additional file 1: Fig. S1.** The 97 prenatal cases with clear clinical results studied herein subdivided by normal and abnormal phenotypes. The first column all 97 cases (14/22 – all) are depicted, in the second and third columns include only sSMCs derived from chromosomes 14 or 22, i.e. der(14) and der(22), respectively; the last column includes the unresolvable cases (14/22 – unres). Normal cases are highlighted in blue, abnormal in red.

**Additional file 2: Tables S1, S2, S3.**

## Data Availability

All data generated or analyzed during this study are included in this published article and its supplementary information files.

## Abbreviations

FISH: Fluorescence in situ hybridization
isoUPD: Isodisomy
OMIM: Online Mendelian Inheritance in Man
sSMCs: Small supernumerary marker chromosomes
sSMCs(14 or 22): SSMC derived from chromosome 14 or 22
UPD: Uniparental disomy
UPD(14): UPD of chromosomes 14
UPD(22): UPD of chromosomes 22
