# Supplementary figures and images for: Small supernumerary marker chromosomes derived from chromosome 14 and/or 22
Source: Mol Cytogenet. 2021 Feb 25;14:13. doi: 10.1186/s13039-021-00533-6 (PMC7908736; doi:10.1186/s13039-021-00533-6)

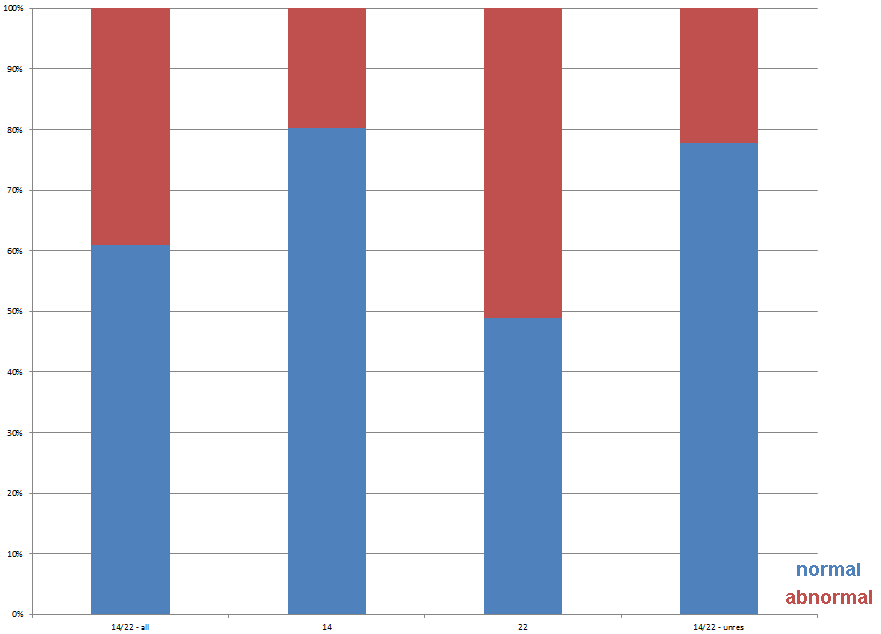

Supplement: Supplementary file 1 — Additional file 1: Fig. S1. The 97 prenatal cases with clear clinical results studied herein subdivided by normal and abnormal phenotypes. The first column all 97 cases (14/22 – all) are depicted, in the second and third columns include only sSMCs derived from chromosomes 14 or 22, i.e. der(14) and der(22), respectively; the last column includes the unresolvable cases (14/22 – unres). Normal cases are highlighted in blue, abnormal in red. [file 13039_2021_533_MOESM1_ESM.tif]
