# Supplementary material for: Small supernumerary marker chromosomes derived from chromosome 14 and/or 22
Source: Mol Cytogenet. 2021 Feb 25;14:13. doi: 10.1186/s13039-021-00533-6 (PMC7908736; doi:10.1186/s13039-021-00533-6)
Supplement: Supplementary file 2 — Additional file 2: Tables S1, S2, S3. [file 13039_2021_533_MOESM2_ESM.docx]

Additional File

# Tables

Abbreviations (apart from ISCN nomenclature):

AF = amnion; CH = chorion; FISH = fluorescence in situ hybridization; m = months; Mat = maternal; MB = megabasepair / genomic position; n.a. = not available; n.y.p. = not yet published, Pat = paternal; PBL = peripheral blood, w= weeks; y = years

**Table S1a**

Small supernumerary marker chromosomes derived from chromosome 14 or 22 without associated clinical signs – only cases from the authors’ laboratory.

Case numbers acc. to sSMC database <http://cs-tl.de/DB/CA/sSMC/0-Start.html>
References acc. to <http://cs-tl.de/DB/CA/sSMC/14-22/z-Ref.html>
Additional case details can be found at <http://cs-tl.de/DB/CA/sSMC/14-22/b-norm.html>

| **case no.** | **gender/ age at diagnosis** | **studied material** | **de novo/ inherited** | **GTG-banding result grade of mosaicism** | **final result of the sSMC** | [**Reference**](http://cs-tl.de/DB/CA/sSMC/14-22/z-Ref.html) |
| --- | --- | --- | --- | --- | --- | --- |
| **14/22-** **O-** **q11.1/** **1-1** | male/ prenatal | AF | paternal (3/50 mitosis with mar) | 47,XY,+mar[15]/ 46,XY[5] | **min(22)(pter**→**q11.1:) or min(14)(14pter**→**14q11.1)** | n.y.p. |
| **14/22- O- q11.1/ 1-3** | female/ 27y | PBL | n.a. | 47,XX,+mar[24]/ 46,XX[6] | **min(22)(pter**→**q11.1:) or min(14)(14pter**→**14q11.1)** | n.y.p. |
| **14/22-** **O-** **q11.1/** **2-5** | male/ 39y | PBL | n.a. | 47,XY,+mar[100%] | **inv dup(14 or 22)(q11.1)*** | n.y.p. |
| **14/22- O- q11.1/ 2-8** | female/ 42y | PBL | n.a. | 47,XX,+mar[100%] | **inv dup(14 or 22)(q11.1)** | n.y.p. |
| **14/22- O- q11.1/ 2-9** | male/ 33y | PBL | n.a. | 47,XY,+mar[23]/ 46,XY[7] | **inv dup(14 or 22)(q11.1)** | n.y.p. |
| **14/22- O- q11.1/ 2-10** | male/ adult | PBL | n.a. | 47,XY,+mar[24]/ 46,XY[26] | **inv dup(14 or 22)(q11.1)** | n.y.p. |
| **14/22-** **O-** **q11.1/** **3-1** | female/ prenatal | AF | de novo | 49,XX,+3mar[9]/ 48,XX,+2mar[6]/ 46,XX[17] | **all sSMC: inv dup(14 or 22)(q11.1)** | n.y.p. |

**Table S1b**

Small supernumerary marker chromosomes derived from chromosome 14 or 22 with associated clinical signs – only not yet published cases from authors’ laboratory.

Case numbers acc. to sSMC database <http://cs-tl.de/DB/CA/sSMC/0-Start.html>
References acc. to <http://cs-tl.de/DB/CA/sSMC/14-22/z-Ref.html>
Additional case details can be found at <http://cs-tl.de/DB/CA/sSMC/14-22/c-abnorm.html>

| **case no.** | **gender/ age at diagnosis** | **studied material** | **de novo/ inherited** | **GTG-banding result grade of mosaicism** | **final result of the sSMC** | [**Reference**](http://cs-tl.de/DB/CA/sSMC/14-22/z-Ref.html) |
| --- | --- | --- | --- | --- | --- | --- |
| **14/22- W- q11.1/ 1-1** | male/ 18y | PBL | n.a. | 47,XY,+mar[37]/ 46,XY[13] | **min(14)(:p11.1**→**q11.1:) or min(22)(:p11.1**→**q11.1:)** | n.y.p. |
| **14/22-** **CW- 3** | male/ 6y | PBL | n.a. | 47,XY,+mar[?%]/ 46,XY[?%] | **mar(14 or 22)** | n.y.p. |

**Table S1c**

Small supernumerary marker chromosomes derived from chromosome 14 or 22 without clear clinical information and/or correlation – only not yet published cases from authors’ laboratory.

Case numbers acc. to sSMC database <http://cs-tl.de/DB/CA/sSMC/0-Start.html>
References acc. to <http://cs-tl.de/DB/CA/sSMC/14-22/z-Ref.html>
Additional case details can be found at <http://cs-tl.de/DB/CA/sSMC/14-22/d-uncl.html>

| **case no.** | **gender/ age at diagnosis** | **studied material** | **de novo/ inherited** | **GTG-banding result grade of mosaicism** | **final result of the sSMC** | [**Reference**](http://cs-tl.de/DB/CA/sSMC/14-22/z-Ref.html) |
| --- | --- | --- | --- | --- | --- | --- |
| **14/22-** **U- 2** | male/ prenatal | AF | de novo | 47,XY,+mar[15] | **dic(22;22)(pter**→**q11.1: :q11.1**→**pter) or dic(14;22)(14pter**→**14q10: :22q11.1**→**22pter)** | n.y.p. |
| **14/22-** **U- 9** | male/ prenatal | AF | de novo | 47,XY,+mar[100%] | **min(22)(pter**→**q11.1:) or min(14)(14pter**→**14q11.1:)** | n.y.p. |
| **14/22-** **U- 16** | female/ prenatal | AF | n.a. | 47,XX,+mar[100%] | **inv dup(14 or 22)(q11.1)** | n.y.p. |
| **14/22-** **U- 20** | male/ prenatal | AF | n.a. | 47,XY,+mar[100%] | **inv dup(14 or 22)(q11.1)** | n.y.p. |
| **14/22- U- 24** | female/ prenatal | AF | n.a. | 47,XX,+mar[100%] | **min(14 or 22)(q11.1)** | n.y.p. |
| **14/22- U- 25** | female/ prenatal | AF | n.a. | 47,XX,+mar[100%] | **inv dup(14 or 22)(q10)** | n.y.p. |
| **14/22- U- 26** | female/ prenatal | AF | n.a. | 47,XX,+mar[100%] | **min(14 or 22)(:p11.1→q11.1:)** | n.y.p. |
| **14/22- U- 27** | female/ 15y | PBL | n.a. | 47,XX,+mar[100%] | **inv dup(14 or 22)(q11.1)** | n.y.p. |
| **14/22- U- 28** | n.a./ adult | PBL | n.a. | 47,XN,+mar[2]/ 46,XN[18] | **min(14 or 22) (:p11.1→q11.1:)** | n.y.p. |
| **14/22- U- 29** | male/ prenatal | AF | n.a. | 47,XY,+mar[16]/ 46,XY[22] | **min(14 or 22) (:p11.1→q11.1:)** | n.y.p. |

**Table S2a**

Small supernumerary marker chromosomes derived from chromosome 14 without any associated clinical signs – only cases from authors’ laboratory.

Case numbers acc. to sSMC database <http://cs-tl.de/DB/CA/sSMC/0-Start.html>
References acc. to <http://cs-tl.de/DB/CA/sSMC/14/z-Ref.html>
Additional case details can be found at <http://cs-tl.de/DB/CA/sSMC/14/b-norm.html>

| **case no.** | **gender/ age at diagnosis** | **studied material** | **de novo/ inherited** | **GTG-banding result grade of mosaicism** | **final result of the sSMC** | [**Reference**](http://cs-tl.de/DB/CA/sSMC/14/z-Ref.html) |
| --- | --- | --- | --- | --- | --- | --- |
| **14- O-** **q10/** **1-1** | male/ prenatal | AF | de novo | 47,XY,+mar[10] | **inv dup(14)(q10)** | [{1} case 20](http://cs-tl.de/DB/CA/sSMC/14/z-Ref.html) |
| **14- O-** **q10/** **1-2** | male/ 27y | PBL | de novo | 47,XY,+mar[15] | **inv dup(14)(q10)** | [{31} case 14-4^2^ {45} case 25 {78} case 14-1](http://cs-tl.de/DB/CA/sSMC/14/z-Ref.html) |
| **14- O-** **q10/** **1-3** | male/ 28y | PBL | n.a. | 47,XY,+mar[15] | **inv dup(14)(q10)** | [{31} case 14-4^3^ {45} case 26 {78} case 14-2](http://cs-tl.de/DB/CA/sSMC/14/z-Ref.html) |
| **14- O-** **q10/** **1-4** | male/ 34y | PBL | n.a. | 47,XY,+mar[15] | **inv dup(14)(q10)** | [{31} case 14-4^4^ {45} case 27 {78} case 14-3](http://cs-tl.de/DB/CA/sSMC/14/z-Ref.html) |
| **14- O-** **q10/** **1-5** | male/ 38y | PBL | n.a. | 47,XY,+mar[15] | **inv dup(14)(q10)** | [{31} case 14-4^5^](http://cs-tl.de/DB/CA/sSMC/14/z-Ref.html) |
| **14- O-** **q10/** **1-14** | male/ adult | PBL | n.a. | 47,XY,+mar[15] | **inv dup(14)(q10)** | [{31} case 14-4^14^ {45} case 29 {78} case 14-4](http://cs-tl.de/DB/CA/sSMC/14/z-Ref.html) |
| **14- O-** **q10/** **1-15** | female/ adult | PBL | n.a. | 47,XX,+mar[15] | **inv dup(14)(q10)** | [{31} case 14-4^15^](http://cs-tl.de/DB/CA/sSMC/14/z-Ref.html) |
| **14- O-** **q10/** **1-16** | female/ 38y | PBL | n.a. | 47,XX,+mar[100%] | **inv dup(14)(q10)** | [{31} case 14-4^16^](http://cs-tl.de/DB/CA/sSMC/14/z-Ref.html) |
| **14- O-** **q11/** **1-1** | male/ prenatal | AF | maternal (age 34y) | 47,XY,+mar[15] | **inv dup(14)(q11)** | [{3} case 3](http://cs-tl.de/DB/CA/sSMC/14/z-Ref.html) |
| **14- O-** **q11/** **1-2** | female/ 28y | PBL | n.a. | 47,XX,+mar[50%]/ 46,XX[50%] | **inv dup(14)(q11)** | [{31} case 14-6](http://cs-tl.de/DB/CA/sSMC/14/z-Ref.html) |
| **14- O-** **q11.1/** **1-1** | female/ 37y | PBL | n.a. | 47,XX,+mar[43]/ 46,XX[7] | **inv dup(14)(q11.1)** | [{31} case 14-7 {45} case 30 {78} case 14-5](http://cs-tl.de/DB/CA/sSMC/14/z-Ref.html) |

**Table S2a (ctd.)**

| **case no.** | **gender/ age at diagnosis** | **studied material** | **de novo/ inherited** | **GTG-banding result grade of mosaicism** | **final result of the sSMC** | [**Reference**](http://cs-tl.de/DB/CA/sSMC/14/z-Ref.html) |
| --- | --- | --- | --- | --- | --- | --- |
| **14- O-** **q11.1/** **1-2** | female/ 38y | PBL | n.a. | 47,XX,+mar[100%] | **inv dup(14)(q11.1)** | [{31} case 14-8 {45} case 31 {78} case 14-6](http://cs-tl.de/DB/CA/sSMC/14/z-Ref.html) |
| **14- O-** **q11.1/** **1-3** | male/ 36y | PBL | n.a. | 47,XY,+mar[100%] | **inv dup(14)(q11.1)** | [{45} case 32 {78} case 14-7](http://cs-tl.de/DB/CA/sSMC/14/z-Ref.html) |
| **14- O-** **q11.1/** **1-4** | male/ prenatal | AF | de novo | 47,XY,+mar[100%] | **inv dup(14)(q11.1)** | n.y.p. |
| **14- O-** **q11.1/** **1-6** | male/ 39y | PBL | n.a. | 47,XY,+mar[100%] | **inv dup(14)(q11.1)** | [{45} case 33 {78} case 14-8](http://cs-tl.de/DB/CA/sSMC/14/z-Ref.html) |
| **14- O-** **q11.1/** **1-7** | female/ 25y | PBL | paternal | 47,XX,+mar[100%] | **inv dup(14)(q11.1)** | n.y.p. |
| **14- O-** **q11.1/** **1-8** | male/ adult | PBL | n.a. | 47,XY,+mar[100%] | **inv dup(14)(q11.1)** | [{45} case 34 {47} case 8 {78} case 14-9](http://cs-tl.de/DB/CA/sSMC/14/z-Ref.html) |
| **14- O-** **q11.1/** **1-9** | female/ 32y | PBL | n.a. | 47,XX,+mar[100%] | **inv dup(14)(q11.1)** | n.y.p. |
| **14- O-** **q11.1/** **1-10** | female/ 29y | PBL | n.a. | 47,XX,+mar[52%] 46,XX[48%] | **inv dup(14)(q11.1)** | [{39} case 6 {45} case 35 {78} case 14-10 {78} case 14-11](http://cs-tl.de/DB/CA/sSMC/14/z-Ref.html) |
| **14- O-** **q11.1/** **1-11** | male/ 45y | PBL | n.a. | 47,XY,+mar[100%] | **inv dup(14)(q11.1)** | [{45} case 36](http://cs-tl.de/DB/CA/sSMC/14/z-Ref.html) |
| **14- O-** **q11.1/** **1-12** | female/ adult | PBL | n.a. | 47,XX,+mar[100%] | **inv dup(14)(q11.1)** | [{47} case 9 {78} case 14-12](http://cs-tl.de/DB/CA/sSMC/14/z-Ref.html) |
| **14- O-** **q11.1/** **1-13** | female/ adult | PBL | n.a. | 47,XX,+mar[100%] | **inv dup(14)(q11.1)** | [{47} case 10](http://cs-tl.de/DB/CA/sSMC/14/z-Ref.html) |
| **14- O-** **q11.1/** **1-14** | female/ 27y | PBL | paternal | 47,XX,+mar[100%] | **inv dup(14)(q11.1)** | n.y.p. |
| **14- O- q11.1/ 1-15** | male/ prenatal | AF | maternal | 47,XY,+mar[100%] | **inv dup(14)(q11.1)** | n.y.p. |

**Table S2a (ctd.)**

| **case no.** | **gender/ age at diagnosis** | **studied material** | **de novo/ inherited** | **GTG-banding result grade of mosaicism** | **final result of the sSMC** | [**Reference**](http://cs-tl.de/DB/CA/sSMC/14/z-Ref.html) |
| --- | --- | --- | --- | --- | --- | --- |
| **14- O-** **q11.1/** **1-16** | male/ adult | PBL | n.a. | 47,XY,+mar[100%] | **inv dup(14)(q11.1)** | [{78} case 14-13](http://cs-tl.de/DB/CA/sSMC/14/z-Ref.html) |
| **14- O-** **q11.1/** **1-17** | male/ 35y | PBL | n.a. | 47,XY,+mar[100%] | **inv dup(14)(q11.1)** | [{78} case 14-14](http://cs-tl.de/DB/CA/sSMC/14/z-Ref.html) |
| **14- O-** **q11.1/** **1-18** | female/ prenatal | AF | de novo | 47,XX,+mar[100%] | **inv dup(14)(q11.1)** | [{58} case 19](http://cs-tl.de/DB/CA/sSMC/14/z-Ref.html) |
| **14- O-** **q11.1/** **1-19** | female/ 63y | PBL | n.a. | 47,XX,inv(19)(p13.3q13.1), +mar[100%] | **inv dup(14)(q11.1)** | n.y.p. |
| **14- O-** **q11.1/** **1-20** | female/ prenatal | AF | maternal | 47,XX,+mar[100%] | **inv dup(14)(q11.1)** | n.y.p. |
| **14- O-** **q11.1/** **1-21** | male/ prenatal | AF | de novo | 47,XY,+mar[100%] | **inv dup(14)(q11.1)** | [{58} case 20](http://cs-tl.de/DB/CA/sSMC/14/z-Ref.html) |
| **14- O-** **q11.1/** **1-22** | male/ prenatal | AF | de novo | 47,XY,+mar[96%] 46,XY[4%] | **inv dup(14)(q11.1)** | n.y.p. |
| **14- O-** **q11.1/** **1-24** | male/ prenatal | AF | n.a. | 47,XY,+mar[100%] | **inv dup(14)(q11.1)** | n.y.p. |
| **14- O-** **q11.1/** **1-26** | female/ prenatal | AF | de novo | 47,XX,+mar[100%] | **inv dup(14)(q11.1)** | n.y.p. |
| **14- O-** **q11.1/** **1-27** | female/ 32y | AF | n.a. | 47,XX,+mar[100%] | **inv dup(14)(q11.1)** | n.y.p. |
| **14- O-** **q11.1/** **1-28** | male/ prenatal | AF | maternal | 47,XY,+mar[100%] | **inv dup(14)(q11.1)** | [{58} case 21](http://cs-tl.de/DB/CA/sSMC/14/z-Ref.html) |
| **14- O-** **q11.1/** **1-29** | male/ 33y | PBL | n.a. | 47,XY,+mar[100%] | **inv dup(14)(q11.1)** | [{78} case 14-15](http://cs-tl.de/DB/CA/sSMC/14/z-Ref.html) |

**Table S2a (ctd.)**

| **case no.** | **gender/ age at diagnosis** | **studied material** | **de novo/ inherited** | **GTG-banding result grade of mosaicism** | **final result of the sSMC** | [**Reference**](http://cs-tl.de/DB/CA/sSMC/14/z-Ref.html) |
| --- | --- | --- | --- | --- | --- | --- |
| **14- O-** **q11.1/** **1-30** | female/ prenatal | PBL | maternal | 47,XX,+mar[100%] | **inv dup(14)(q11.1)** | n.y.p. |
| **14- O-** **q11.1/** **1-31** | female/ 36y | PBL | n.a. | 47,XX,+mar[7]/ 46,XX[23] | **inv dup(14)(q11.1)** | [{78} case 14-16](http://cs-tl.de/DB/CA/sSMC/14/z-Ref.html) |
| **14- O-** **q11.1/** **1-32** | female/ 29y | PBL | n.a. | 47,XX,+mar[100%] | **inv dup(14)(q11.1)** | [{78} case 14-17](http://cs-tl.de/DB/CA/sSMC/14/z-Ref.html) |
| **14- O-** **q11.1/** **1-33** | male/ prenatal | AF | n.a. | 47,XY,+mar[61%]/ 46,XY[39%] | **inv dup(14)(q11.1)** | n.y.p. |
| **14- O- q11.1/ 1-34** | female/ prenatal | AF | n.a. | 47,XX,+mar[100%] | **inv dup(14)(q11.1)** | n.y.p. |
| **14- O- q11.1/ 1-35** | male/ 35y | PBL | n.a. | 47,XY,+mar[100%] | **inv dup(14)(q11.1)** | [78} case 14-18](http://cs-tl.de/DB/CA/sSMC/14/z-Ref.html) |
| **14- O- q11.1/ 1-36** | male/ prenatal | AF | pat | 47,XY,+mar[100%] | **inv dup(14)(q11.1)** | n.y.p. |
| **14- O- q11.1/ 1-37** | male/ prenatal | AF | mat | 47,XY,+mar[100%] | **inv dup(14)(q11.1)** | n.y.p. |
| **14- O- q11.1/ 1-38** | male/ 43y | PBL | n.a. | 47,XY,+mar[100%] | **inv dup(14)(q11.1)** | n.y.p. |
| **14- O- q11.1/ 1-39** | male/ adult | PBL | n.a. | 47,XY,+mar[100%] | **inv dup(14)(q11.1)** | n.y.p. |
| **14- O- q11.1/ 1-40** | male/ prenatal | AF | n.a. | 47,XY,+mar[~90%]/ 46,XY[~10%] | **inv dup(14)(q11.1)** | n.y.p. |
| **14- O- q11.1/ 1-41** | male/ 32y | PBL | n.a. | 47,XY,+mar[16]/ 46,XY[84] | **inv dup(14)(q11.1)** | n.y.p. |
| **14- O- q11.1/ 1-42** | male/ 46y | PBL | n.a. | 47,XY,+mar[6]/ 46,XY[14] | **inv dup(14)(q11.1)** | n.y.p. |

**Table S2a (ctd.)**

| **case no.** | **gender/ age at diagnosis** | **studied material** | **de novo/ inherited** | **GTG-banding result grade of mosaicism** | **final result of the sSMC** | [**Reference**](http://cs-tl.de/DB/CA/sSMC/14/z-Ref.html) |
| --- | --- | --- | --- | --- | --- | --- |
| **14- O- q11.1/ 1-43** | female/ 39y | PBL | n.a. | 47,XX,+mar[100%] | **inv dup(14)(q11.1)** | n.y.p. |
| **14- O- q11.1/ 1-44** | female/ 36y | PBL | n.a. | 47,XX,+mar[100%] | **inv dup(14)(q11.1)** | n.y.p. |
| **14- O- q11.1/ 1-45** | male/ 17y | PBL | n.a. | 47,XY,+mar[100%] | **inv dup(14)(q11.1)** | n.y.p. |
| **14- O- q11.1/ 1-46** | female/ 33y | PBL | n.a. | 47,XX,+mar[100%] | **inv dup(14)(q11.1)** | n.y.p. |
| **14- O- q11.1/ 1-47** | male/ 30y | PBL | n.a. | 47,XY,+mar[100%] | **inv dup(14)(q11.1)** | n.y.p. |
| **14- O- q11.1/ 1-48** | male/ adult | PBL | n.a. | 47,XY,+mar[100%] | **inv dup(14)(q11.1)** | n.y.p. |
| **14- O- q11.1/ 1-49** | female/ 35y | PBL | n.a. | 47,XX,+mar[100%] | **inv dup(14)(q11.1)** | n.y.p. |
| **14- O- q11.1/ 1-50** | male/ 42y | PBL | n.a. | 47,XY,+mar[100%] | **inv dup(14)(q11.1)** | n.y.p. |
| **14- O- q11.1/ 1-51** | female/ 41y | PBL | n.a. | 47,XX,+mar[100%] | **inv dup(14)(q11.1)** | n.y.p. |
| **14- O- q11.1/ 1-52** | female/ 30y | PBL | n.a. | 47,XX,+mar[100%] | **inv dup(14)(q11.1)** | n.y.p. |
| **14- O- q11.1/ 1-53** | female/ 34y | PBL | n.a. | 47,XX,+mar[100%] | **inv dup(14)(q11.1)** | n.y.p. |
| **14- O- q11.1/ 1-54** | male/ 4m | PBL | pat | 47,XY,+mar[100%] | **inv dup(14)(q11.1)** | n.y.p. |
| **14- O- q11.1/ 1-55** | female/ prenatal | AF | pat | 47,XX,+mar[100%] | **inv dup(14)(q11.1)** | n.y.p. |

**Table S2a (ctd.)**

| **case no.** | **gender/ age at diagnosis** | **studied material** | **de novo/ inherited** | **GTG-banding result grade of mosaicism** | **final result of the sSMC** | [**Reference**](http://cs-tl.de/DB/CA/sSMC/14/z-Ref.html) |
| --- | --- | --- | --- | --- | --- | --- |
| **14- O- q11.1/ 1-56** | male/ 34y | PBL | n.a. | 47,XY,+mar[100%] | **inv dup(14)(q11.1)** | n.y.p. |
| **14- O- q11.1/ 1-57** | male/ 28y | PBL | n.a. | 47,XY,+mar[100%] | **inv dup(14)(q11.1)** | n.y.p. |
| **14- O- q11.1/ 1-58** | female/ 19y | PBL | n.a. | 47,XX,+mar[23]/ 46,XX[7] | **inv dup(14)(q11.1) aCGH: no result - and false positive on pericentr. 12** | n.y.p. |
| **14- O- q11.1/ 1-59** | female/ 38y | PBL | n.a. | 47,XX,+mar[100%] | **inv dup(14)(q11.1)** | n.y.p. |
| **14- O- q11.1/ 1-60** | male/ 36y | PBL | n.a. | 47,XY,+mar[12]/ 46,XY[18] | **inv dup(14)(q11.1)** | n.y.p. |
| **14- O-** **q11.1/** **2-1** | male/ prenatal | CH; umbilical chord blood (UBL) | de novo | CH: 47,XY,+mar[50%]/ 46,XY[50%] UBL: 48,XY,+marx2[2%]/ 47,XY+mar[12]/ 46,XY[3] | **inv dup(14)(q11.1)x2** | n.y.p. |
| **14- O-** **q11.1/** **2-2** | female/ 40y | PBL | n.a. | 48,XX,+2mar[100%] | **inv dup(14)(q11.1)x2** | [{45} case 37 {78} case 14-19](http://cs-tl.de/DB/CA/sSMC/14/z-Ref.html) |
| **14- O- q11.1/ 3-1** | male/ 37y | PBL | n.a. | 47,XY,t(5;6)(q21;q12),+mar[100%] | **inv dup(14)(q11.1)** | n.y.p. |
| **14- O-** **q11.1/** **4-1** | female/ prenatal | AF | de novo | 47,XX,+mar[100%] | **min(14)(:p11.1**→**q11.1:) or min(14)(pter**→**q11.1:)** | n.y.p. |
| **14- O-** **q11.1/** **4-2** | male/ prenatal | AF | n.a. | 47,XY,+mar[100%] | **min(14)(pter**→**q11.1:)** | [{70} case Sm-6](http://cs-tl.de/DB/CA/sSMC/14/z-Ref.html) |
| **14- O- q11.1/ 4-3** | male/  adult | PBL | n.a. | 47,XY,+mar[100%] | **min(14)(pter**→**q11.1:)** | n.y.p. |
| **14- O- q11.1/ 4-4** | female/  35y | PBL | n.a. | 47,XX,+mar[14]/ 46,XX[36] | **min(14)(pter**→**q11.1:)** | n.y.p. |
| **14- O- q11.1/ 5-1** | male/ 50y | PBL | n.a. | 47,XY,+mar[7]/ 46,XY[13] | **r(14)(::p13**→**q11.1::),cen+** | [{78} case 14-21](http://cs-tl.de/DB/CA/sSMC/14/z-Ref.html) |

**Table S2a (ctd.)**

| **case no.** | **gender/ age at diagnosis** | **studied material** | **de novo/ inherited** | **GTG-banding result grade of mosaicism** | **final result of the sSMC** | [**Reference**](http://cs-tl.de/DB/CA/sSMC/14/z-Ref.html) |
| --- | --- | --- | --- | --- | --- | --- |
| **14- O-** **q11.1/** **6-1** | female/ 40y | PBL | n.a. | 47,XX,+mar[15%]/ 46,XX[85%] | **r(14)(::p1?2**→**q11.1::)** | n.y.p. |
| **14- O-** **q11.2/** **2-1** | male/ prenatal | AF | n.a. | 47,XY+mar[15]/ 46,XY[10] | **dic(14)(:p11.1**→**q11.1: :p11.1**→**q11.2:) BAC** **RP11-324B11** **in 14q11.2 present once (19.88MB)** | n.y.p. |
| **14- O-** **q11.2/** **3-1** | female/ 29y | PBL | n.a. | 47,XX,+mar[40]/ 46,XX[60] | **min(14)(pter**→**q11.2:) BAC** **RP11-324B11** **in 14q11.2 present once (19.88MB)** | [{45} case 38 {78} case 14-22](http://cs-tl.de/DB/CA/sSMC/14/z-Ref.html) |
| **14- O-** **q11.2/** **4-1** | male/ 32y | PBL | n.a. | 47,XY,+mar[100%] | **r(14)(::p11.2**→**q11.2::)** | [{42} {78} case 14-23](http://cs-tl.de/DB/CA/sSMC/14/z-Ref.html) |
| **14- O-** **q11.2/** **5-1** | male/ prenatal | AF | maternal (in PBL sSMC 100%) | 47,XY,+mar[100%] | **inv dup(14)(q11.2) BAC** **RP11-324B11** **in 14q11.2 present twice (19.88MB) array: 18.45-20.24** | [{70} case Si-1](http://cs-tl.de/DB/CA/sSMC/14/z-Ref.html) |
| **14-** **CO- 18** | female/ 30y | PBL | n.a. | 47,XX,+mar[100%] | **mar(14)** | n.y.p. |

**Table S2b**

Small supernumerary marker chromosomes derived from chromosome 14 with associated clinical signs – only not yet published cases from authors’ laboratory.

Case numbers acc. to sSMC database <http://cs-tl.de/DB/CA/sSMC/0-Start.html>
References acc. to <http://cs-tl.de/DB/CA/sSMC/14/z-Ref.html>
Additional case details can be found at <http://cs-tl.de/DB/CA/sSMC/14/c-abnorm.html>

| **case no.** | **gender/ age at diagnosis** | **studied material** | **de novo/ inherited** | **GTG-banding result grade of mosaicism** | **final result of the sSMC** | [**Reference**](http://cs-tl.de/DB/CA/sSMC/14/z-Ref.html) |
| --- | --- | --- | --- | --- | --- | --- |
| **14- W-** **q10/** **1-1** | female/ prenatal | AF | n.a. | 47,XX,+mar[95%]/ 46,XY[5%] | **inv dup(14)(q10)** | n.y.p. |
| **14- W-** **q10/** **1-2** | female/ prenatal | AF | n.a. | 47,XX,+mar[%?] | **inv dup(14)(q10)** | n.y.p. |
| **14- W-** **q11.2/** **2-2** | male/ 16y | PBL | n.a. | 47,XY,+mar[14]/ 46,XY[16] | **r(14)(::p11.?2**→**q11.2::)** | n.y.p. |
| **14- W-** **q13/** **2-1** | female/ prenatal | AF | de novo | 47,XX,+mar[100%] | **min(14)(:q13**→**q24.3:)** | n.y.p. |
| **14- W-** **q13/** **3-1** | female/ newborn | PBL | n.a. | 47,XX,+mar[100%] | **min(14)(pter**→**q13:)** | n.y.p. |
| **14- W-** **q13.3/** **1-1** | male/ 17y | PBL | de novo | 47,XY,inv(9)(p12q13),+mar[40%]/ 46,XY,inv(9)(p12q13)[60%] | **r(14)(::p12**→**q13.3::)** | n.y.p. |
| **14- W-** **q21.2**/ **1-2** | male/ 5y | PBL | n.a. | 47,XY,+mar[100%] | **min(14)(pter**→**q21.1:)** | n.y.p. |
| **14- W-** **q21.3**/ **1-1** | male/ 1y | PBL | n.a. | 47,XY,+mar[100%] | **min(14)(pter**→**q21.2:)** | n.y.p. |
| **14- Uc- 1** | male/ prenatal | AF | de novo | 46,XY,der(13;14), +mar[100%] | **46,XX,t(13;14)(p11.2;p11.1) can be an sSMC in next generation** | n.y.p. |
| **14- Uc- 4** | female/ postnatal | PBL | n.a. | 47,XX,+mar,16qh-[19]/ 46,XX,16qh-[1] | **der(14)t(5;14)(p13.3;q13)** | [{72; 73}](http://cs-tl.de/DB/CA/sSMC/14/z-Ref.html) |
| **14- Uc- 7** | male/ 5y | PBL | maternal t(14;17) | 47,XY,+mar[100%] | **der(14)t(14;17)(q11.2;q25.3)** | [{73}](http://cs-tl.de/DB/CA/sSMC/14/z-Ref.html) |
| **14- Uc- 10** | male/ 3y | PBPBLL | n.a. | 47,XY,+mar[100%] | **der(14)t(8;14)(p23;q22)** | [{73; 76}](http://cs-tl.de/DB/CA/sSMC/14/z-Ref.html) |

**Table S2b (ctd.)**

| **case no.** | **gender/ age at diagnosis** | **studied material** | **de novo/ inherited** | **GTG-banding result grade of mosaicism** | **final result of the sSMC** | [**Reference**](http://cs-tl.de/DB/CA/sSMC/14/z-Ref.html) |
| --- | --- | --- | --- | --- | --- | --- |
| **14- Uc- 12** | female/ postnatal | PBL | mat (balanced in mother) | 47,XY,+mar[100%] | **der(14)t(6;14)(p25;q11.1~11.2)** | [{73} cases 10 and 11](http://cs-tl.de/DB/CA/sSMC/14/z-Ref.html) |
| **14- Uc- 13** | female/ prenatal | AF | de novo | 46,XX,t(13;14),+mar[100%] | **46,XX,t(13;14)(p11.2;p11.1)dn** | n.y.p. |
| **14- Uc- 14** | male/ 30y | PBL | n.a. | 46,XY,t(14;21),+mar | **46,XY,t(14;21)(q11.1~11.2;p13)** | n.y.p. |
| **14- Uc- 17** | female/ 3y | PBL | mat (balanced t(1;14) in mother; she also has partial trisomy 5q35.3 to 5qter | 47,XY,+mar[100%] | **der(14)(5qter→5q35.3: :14p1?3**→**14q13: 1q44→1qter)** | [{0}](http://cs-tl.de/DB/CA/sSMC/14/z-Ref.html) |
| **14- Uu- 1** | male/ prenatal | AF | de novo | 47,XY,+mar[100%] | **mar(14)** maternal UPD | [{35} case 7](http://cs-tl.de/DB/CA/sSMC/14/z-Ref.html) |
| **14- Uu- 3** | male/ 31y | PBL | de novo | 47,XY,+mar[26]/ 46,XY[4] | **min(14)(pter**→**q11.1:)** maternal UPD 14 | [{72}](http://cs-tl.de/DB/CA/sSMC/14/z-Ref.html) |
| **14- Uu- 4** | male/ prenatal | AF and PBL | de novo | 47,XY,+mar[100%] | **min(14)(pter**→**q11.1~q11.2:)** maternal UPD 14 | {35} case 3 |

**Table S2c**

Small supernumerary marker chromosomes derived from chromosome 14 without clear clinical information and/or correlation – only not yet published cases from authors’ laboratory.

Case numbers acc. to sSMC database <http://cs-tl.de/DB/CA/sSMC/0-Start.html>
References acc. to <http://cs-tl.de/DB/CA/sSMC/14/z-Ref.html>
Additional case details can be found at <http://cs-tl.de/DB/CA/sSMC/14/d-uncl.html>

| **case no.** | **gender/ age at diagnosis** | **studied material** | **de novo/ inherited** | **GTG-banding result grade of mosaicism** | **final result of the sSMC** | [**Reference**](http://cs-tl.de/DB/CA/sSMC/14/z-Ref.html) |
| --- | --- | --- | --- | --- | --- | --- |
| **14-** **U- 1** | male/ prenatal | AF | de novo | 47,XY,+mar[5]/ 46,XY[5] | **inv dup(14)(q11)** | [{2} case 4 {4} case 4](http://cs-tl.de/DB/CA/sSMC/14/z-Ref.html) |
| **14-** **U- 1a** | female/ prenatal | AF | de novo | 47,XY,+mar[9]/ 46,XY[5] | **inv dup(14)(q11.1)** | [{2} case 1 {4} case 1](http://cs-tl.de/DB/CA/sSMC/14/z-Ref.html) |
| **14-** **U- 1b** | female/ prenatal | AF | de novo (?) | 47,XX,t(9;14)(q22.1~q22.2;q21), +mar[100%] | **inv dup(14)(q11.1)** | n.y.p. |
| **14-** **U- 1c** | n.a./ prenatal | AF | n.a. | 47,XX,+mar[100%] | **inv dup(14)(q11.1)** | n.y.p. |
| **14-** **U- 1d** | female/ prenatal | AF | n.a. | 47,XX,+mar[28]/ 46,XX[34] | **inv dup(14)(q11.1)** | n.y.p. |
| **14-** **U- 1e** | male/ prenatal | AF | n.a. | 47,XY,+mar[42]/ 46,XY[34] | **inv dup(14)(q11.1)** | n.y.p. |
| **14-** **U- 1f** | male/ prenatal | AF | n.a. | 47,XY,+mar[100%] | **inv dup(14)(q11.1)** | n.y.p. |
| **14-** **U- 2** | female/ 1y | PBL | de novo | 47,XY,+mar[15] | **min(14)(:p12**→**q11.1:)** | n.y.p. |
| **14-** **U- 2a** | male/ postnatal | PBL | n.a. | 47,XY,+mar[50%]/ 46,XY[50%] | **min(14)(:p12**→**q11.1:)** | n.y.p. |
| **14-** **U- 2c** | male/ 1y | PBL | n.a. | 47,XY,+mar[44]/ 46,XY[6] | **min(14)(pter**→**q11.1:)** | n.y.p. |
| **14-** **U- 2e** | male/ prenatal | AF | n.a. | 47,XY,+mar[7]/ 46,XY[17] in interphase sSMC in 70% | **min(14)(p11.1**→**q11.1:)** | n.y.p. |
| **14-** **U- 3** | male/ prenatal | AF | de novo | 47,XY,+mar[50%]/ 46,XY[50%] | **min(14)(:p11.1**→**q11.1:)[7]/ inv dup(14)(:p11.1**→**q11.1: :q11.1**→**p11.1:)[4]** | n.y.p. |
| **14- U- 19** | male/ n.a. | PBL/ EBV line | n.a. | 47,XY,+mar[27]/ 46,XY[3] | **mar(14)(pterq23.33)** | [{65; 73}](http://cs-tl.de/DB/CA/sSMC/14/z-Ref.html) |
| **14- U- 20** | female/ 12y | PBL | n.a. | 47,XX,+mar[100%] | **min(14)(pter**→**q11.1:)** | n.y.p. |

**Table S2c (ctd.)**

| **case no.** | **gender/ age at diagnosis** | **studied material** | **de novo/ inherited** | **GTG-banding result grade of mosaicism** | **final result of the sSMC** | [**Reference**](http://cs-tl.de/DB/CA/sSMC/14/z-Ref.html) |
| --- | --- | --- | --- | --- | --- | --- |
| **14- U- 22** | female/ 2y | PBL | n.a. | 47,XX,+mar[100%] | **inv dup(14)(q11.1)** | n.y.p. |
| **14- U- 24** | male/ 3y | PBL | de novo | 47,XY,+mar[10%]/ 46,XY[90%] | **min(14)(pter**→**q11.2:)** | n.y.p. |
| **14- U- 25** | male/ 14y | PBL | n.a. | 47,XY,+mar[17]/ 46,XY[3] | **inv dup(14)(q11.1)** | n.y.p. |
| **14- U- 29** | female/ ?postnatal | ?PBL | n.a. | 47,XX,+mar[19]/ 46,XX[9] | **min(14)(pter**→**q11.1:)** | n.y.p. |
| **14- U- 30** | female/ prenatal | AF | de novo | 47,XX,+mar[100%] | **inv dup(14)(q11.1)** | n.y.p. |
| **14- U- 31** | female/ postnatal | PBL | n.a. | 47,XX,+mar[100%] | **inv dup(14)(q11.1)** | n.y.p. |
| **14- U- 36** | male/ prenatal | AF | de novo | 47,XY,+mar[11]/ 46,XY[4] | **min(14)(pter**→**q11.1:) or min(14)(:p11.1**→**q11.1:)** | n.y.p. |
| **14- U- 37** | male/ prenatal | AF | n.a. | 47,XY,+mar[100%] | **inv dup(14)(q11.1)** | n.y.p. |
| **14- U- 40** | male/ prenatal | AF | n.a. | 47,XY,+mar[100%] | **inv dup(14)(q13.2)** | n.y.p. |
| **14- U- 41** | male/ prenatal | AF | n.a. | 47,XY,+mar[11]/ 46,XY[19] | **min(14)(:p11.1→q11.1:)** | n.y.p. |
| **14- U- 43** | male/ 7y | PBL | n.a. | 47,XY,+mar[8]/ 46,XY[202] | **inv dup(14)(q11.1)** | n.y.p. |
| **14- U- 44** | female/ prenatal | AF | n.a. | 47,XX,+mar[100%] | **inv dup(14)(q11.1)** | n.y.p. |
| **14- U- 45** | female/ prenatal | AF | n.a. | 47,XX,+mar[100%] | **inv dup(14)(q11.1)** | n.y.p. |
| **14- U- 46** | female/ prenatal | CH | n.a. | 47,XX,+mar[40%]/ 46,XX[60%] | **inv dup(14)(q11.1)** | n.y.p. |
| **14- U- 47** | female/ prenatal | CH | de novo | 47,XX,+mar[100%] | **inv dup(14)(q11.1)** | n.y.p. |

**Table S3a**

Small supernumerary marker chromosomes derived from chromosome 22 without any associated clinical signs – only cases from authors’ laboratory.

Case numbers acc. to sSMC database <http://cs-tl.de/DB/CA/sSMC/0-Start.html>
References acc. to <http://cs-tl.de/DB/CA/sSMC/22/z-Ref.html>
Additional case details can be found at <http://cs-tl.de/DB/CA/sSMC/22/b-norm.html>

| **case no.** | **gender/ age at diagnosis** | **studied material** | **de novo/ inherited** | **GTG-banding result grade of mosaicism** | **final result of the sSMC** | [**Reference**](http://cs-tl.de/DB/CA/sSMC/22/z-Ref.html) |
| --- | --- | --- | --- | --- | --- | --- |
| **22- O-** **q10/** **1-7** | female / adult | PBL | n.a. | 47,XX,+mar[100%] | **inv dup(22)(q10)** | n.y.p.. |
| **22- O-** **q10/** **1-14** | female / adult | PBL | n.a. | 47,XX,+mar[10%]; in oocytes 40% | **inv dup(22)(q10)** | n.y.p. |
| **22- O-** **q10/** **1-15** | female / 30y | PBL | n.a. | 47,XX,+mar[22]/ 46,XX[28] | **inv dup(22)(q10)** | n.y.p. |
| **22- O-** **q11/** **2-1** | female/ 1y | PBL | de novo | 47,XX,+mar[11]/ 46,XX[39] | **min(22)(:p11.1**→**q11:)** maternal UPD 22 | [{6, 288}](http://cs-tl.de/DB/CA/sSMC/22/z-Ref.html) |
| **22- O-** **q11.1/** **1-1** | female/ prenatal | AF | de novo | 47,XX,+mar[10] Postnatal: 47,XX, +mar[13]/ 46,XX[17] | **inv dup(22)(q11.1)** | [{1} case 31](http://cs-tl.de/DB/CA/sSMC/22/z-Ref.html) |
| **22- O-** **q11.1/** **1-2** | female/ 3m | PBL | de novo | 47,XX,+mar[15] | **inv dup(22)(q11.1)** | [{181} case 22-9](http://cs-tl.de/DB/CA/sSMC/22/z-Ref.html) |
| **22- O-** **q11.1/** **1-3** | male/ prenatal | AF | n.a. | 47,XY,+mar[30%]/ 46,XY[70%] | **inv dup(22)(q11.1)** | n.y.p. |
| **22- O-** **q11.1/** **1-4** | male/ prenatal | AF | n.a. | 47,XY,+mar[12]/ 46,XY[16] | **inv dup(22)(q11.1)** | n.y.p. |
| **22- O-** **q11.1/** **1-5** | male/ 8m | PBL | Mat | 47,XY,+mar[100%] | **inv dup(22)(q11.1)** **in array no imbalance detected** | [{283} case Si-4](http://cs-tl.de/DB/CA/sSMC/22/z-Ref.html) |
| **22- O-** **q11.1/** **1-6** | female/ 32y | PBL | n.a. | 47,XX,+mar[100%] | **inv dup(22)(q11.1)** | [{212} case 101 {321} case 22-2](http://cs-tl.de/DB/CA/sSMC/22/z-Ref.html) |
| **22- O-** **q11.1/** **1-7** | female/ prenatal | AF | n.a. | 47,XX,+mar[100%] | **inv dup(22)(q11.1)** | n.y.p. |

**Table S3a (ctd.)**

| **case no.** | **gender/ age at diagnosis** | **studied material** | **de novo/ inherited** | **GTG-banding result grade of mosaicism** | **final result of the sSMC** | [**Reference**](http://cs-tl.de/DB/CA/sSMC/22/z-Ref.html) |
| --- | --- | --- | --- | --- | --- | --- |
| **22- O-** **q11.1/** **1-8** | male/ 28y | PBL | n.a. | 47,XY,+mar[64% or 81%]/ 46,XY[36% or 19%] | **inv dup(22)(q11.1)** | [{212} case 102 {321} case 22-3](http://cs-tl.de/DB/CA/sSMC/22/z-Ref.html) |
| **22- O-** **q11.1/** **1-9** | female/ 30y | PBL | Mat | 47,XX,+mar[60]/ 46,XX[2] | **inv dup(22)(q11.1)** | n.y.p. |
| **22- O-** **q11.1/** **1-10** | female/ 24y | PBL | n.a. | 47,XX,+mar[100%] | **inv dup(22)(q11.1)** | [{212} case 103 {321} case 22-4](http://cs-tl.de/DB/CA/sSMC/22/z-Ref.html) |
| **22- O-** **q11.1/** **1-11** | female/ 32y | PBL | n.a. | 47,XX,+mar[100%] | **inv dup(22)(q11.1)** | n.y.p. |
| **22- O-** **q11.1/** **1-12** | female/ 27y | PBL | n.a. | 47,XX,+mar[100%] | **inv dup(22)(q11.1)** | [{216} case 33](http://cs-tl.de/DB/CA/sSMC/22/z-Ref.html) |
| **22- O-** **q11.1/** **1-13** | male/ 35y | PBL | n.a. | 47,XY,+mar[100%] | **inv dup(22)(q11.1)** | [{321} case 22-5](http://cs-tl.de/DB/CA/sSMC/22/z-Ref.html) |
| **22- O-** **q11.1/** **1-14** | male/ prenatal | AF, PBL | Pat | 47,XY,+mar[100%] | **inv dup(22)(q11.1)** | n.y.p. |
| **22- O-** **q11.1/** **1-15** | female/ adult | PBL | n.a. | 47,XX,+mar[100%] | **inv dup(22)(q11.1)** | n.y.p. |
| **22- O-** **q11.1/** **1-16** | female/ adult | PBL | n.a. | 47,XX,inv(2)(q13q36), +mar[100%] | **inv dup(22)(q11.1)** | n.y.p. |
| **22- O-** **q11.1/** **1-17** | male/ 40y | PBL | n.a. | 47,XY,+mar[100%] | **inv dup(22)(q11.1)** | [{247} case 15 {321} case 22-6](http://cs-tl.de/DB/CA/sSMC/22/z-Ref.html) |
| **22- O-** **q11.1/** **1-18** | male/ prenatal | AF | n.a. | 47,XY,+mar[6]/ 46,XY[4] | **inv dup(22)(q11.1)** | n.y.p. |
| **22- O-** **q11.1/** **1-19** | female/ prenatal | AF | Pat | 47,XX,+mar[100%] | **inv dup(22)(q11.1)** | n.y.p. |

**Table S3a (ctd.)**

| **case no.** | **gender/ age at diagnosis** | **studied material** | **de novo/ inherited** | **GTG-banding result grade of mosaicism** | **final result of the sSMC** | [**Reference**](http://cs-tl.de/DB/CA/sSMC/22/z-Ref.html) |
| --- | --- | --- | --- | --- | --- | --- |
| **22- O-** **q11.1/** **1-20** | n.a./ postnatal | PBL | n.a. | 47,+mar[100%] | **inv dup(22)(q11.1)** | [{321} case 22-7](http://cs-tl.de/DB/CA/sSMC/22/z-Ref.html) |
| **22- O-** **q11.1/** **1-21** | female/ 32y | PBL | n.a. | 47,XX,+mar[100%] | **inv dup(22)(q11.1)** | [{321} case 22-8](http://cs-tl.de/DB/CA/sSMC/22/z-Ref.html) |
| **22- O-** **q11.1/** **1-22** | female/ adult | PBL | Mat | 47,XX,+mar[100%] | **inv dup(22)(q11.1)** | [{321} case 22-9](http://cs-tl.de/DB/CA/sSMC/22/z-Ref.html) |
| **22- O-** **q11.1/** **1-23** | male/ prenatal | PBL | Mat | 47,XY,+mar[100%] | **inv dup(22)(q11.1)** | n.y.p. |
| **22- O-** **q11.1/** **1-24** | female/ 20y | PBL | Pat | 47,XX,+mar[100%] | **inv dup(22)(q11.1)** | n.y.p. |
| **22- O-** **q11.1/** **1-25** | male/ 2y | PBL | Mat and grand-pat | 47,XY,+mar[100%] | **inv dup(22)(q11.1)** | [{235}](http://cs-tl.de/DB/CA/sSMC/22/z-Ref.html) |
| **22- O-** **q11.1/** **1-26** | male/ prenatal | AF | n.a. | 47,XY,+mar[50%]/ 46,XY[50%] | **inv dup(22)(q11.1)** | n.y.p. |
| **22- O-** **q11.1/** **1-27** | female/ 39y | PBL | n.a. | 47,XX,+mar[100%] | **inv dup(22)(q11.1)** | [{321} case 22-10](http://cs-tl.de/DB/CA/sSMC/22/z-Ref.html) |
| **22- O-** **q11.1/** **1-28** | male/ prenatal | AF | n.a. | 47,XY,+mar[100%] | **inv dup(22)(q11.1)** | n.y.p. |
| **22- O-** **q11.1/** **1-29** | female/ 29y | PBL | n.a. | 47,XX,+mar[100%] | **inv dup(22)(q11.1) array-CGH: 15476855 MB -16042396 MB** | n.y.p. |
| **22- O-** **q11.1/** **1-30** | female/ 32y | PBL | n.a. | 47,XX,+mar[100%] | **inv dup(22)(q11.1)** | [{321} case 22-11](http://cs-tl.de/DB/CA/sSMC/22/z-Ref.html) |
| **22- O-** **q11.1/** **1-31** | male/ prenatal | AF, PBL | Mat | 47,XY,+mar[100%] | **inv dup(22)(q11.1)** | n.y.p. |
| **22- O-** **q11.1/** **1-33** | male/ newborn | AF, PBL | Mat | 47,XY,+mar[100%] | **inv dup(22)(q11.1)** | [{321} case 22-12](http://cs-tl.de/DB/CA/sSMC/22/z-Ref.html) |

**Table S3a (ctd.)**

| **case no.** | **gender/ age at diagnosis** | **studied material** | **de novo/ inherited** | **GTG-banding result grade of mosaicism** | **final result of the sSMC** | [**Reference**](http://cs-tl.de/DB/CA/sSMC/22/z-Ref.html) |
| --- | --- | --- | --- | --- | --- | --- |
| **22- O-** **q11.1/** **1-34** | male/ 40y | PBL | n.a. | 47,XY,+mar[100%] | **inv dup(22)(q11.1)** | [{321} case 22-13](http://cs-tl.de/DB/CA/sSMC/22/z-Ref.html) |
| **22- O-** **q11.1/** **1-36** | male/ adult | PBL | n.a. | 47,XY,+mar[?%]/ 46,XY[?%] | **inv dup(22)(q11.1)** | n.y.p. |
| **22- O-** **q11.1/** **1-37** | male/ prenatal | AF | Mat | 47,XY,+mar[100%] | **inv dup(22)(q11.1)** | [{282} case 1](http://cs-tl.de/DB/CA/sSMC/22/z-Ref.html) |
| **22- O-** **q11.1/** **1-38** | male/ 36y | PBL | n.a. | 47,XY,+mar[100%] | **inv dup(22)(q11.1)** | [{321} case 22-15](http://cs-tl.de/DB/CA/sSMC/22/z-Ref.html) |
| **22- O- q11.1/ 1-42** | female/ adult | PBL | n.a. | 47,XX,+mar[?100%] | **inv dup(22)(q11.1)** | n.y.p. |
| **22- O- q11.1/ 1-43** | female/ prenatal | AF | Mat | 47,XX,+mar[100%] | **inv dup(22)(q11.1)** | n.y.p. |
| **22- O- q11.1/ 1-44** | male/ 38y | PBL | n.a. | 47,XY,+mar[100%] | **inv dup(22)(q11.1)** | [{321} case 22-17](http://cs-tl.de/DB/CA/sSMC/22/z-Ref.html) |
| **22- O- q11.1/ 1-45** | n.a./ prenatal | AF | Mat | 47,XN,+mar[100%] | **inv dup(22)(q11.1)** | n.y.p. |
| **22- O- q11.1/ 1-56** | male/ 42y | PBL | n.a. | 47,XY,+mar[100%] | **inv dup(22)(q11.1)** | [{321} case 22-18](http://cs-tl.de/DB/CA/sSMC/22/z-Ref.html) |
| **22- O- q11.1/ 1-57** | male/ 39y | PBL | n.a. | 47,XY,+mar[100%] | **inv dup(22)(q11.1)** | [{321} case 22-19](http://cs-tl.de/DB/CA/sSMC/22/z-Ref.html) |
| **22- O- q11.1/ 1-58** | female/ 34y | PBL | n.a. | 47,XX,+mar[100%] | **inv dup(22)(q11.1)** | n.y.p. |
| **22- O- q11.1/ 1-60** | n.a./ postnatal | PBL | familial | 47,XN,+mar[100%] | **inv dup(22)(q11.1)** | n.y.p. |
| **22- O- q11.1/ 1-61** | female/ 33y | PBL | n.a. | 47,XX,+mar[100%] | **inv dup(22)(q11.1)** | n.y.p. |

**Table S3a (ctd.)**

| **case no.** | **gender/ age at diagnosis** | **studied material** | **de novo/ inherited** | **GTG-banding result grade of mosaicism** | **final result of the sSMC** | [**Reference**](http://cs-tl.de/DB/CA/sSMC/22/z-Ref.html) |
| --- | --- | --- | --- | --- | --- | --- |
| **22- O- q11.1/ 1-62** | male/ 37y | PBL | n.a. | 47,XY,+mar[100%] | **inv dup(22)(q11.1)** | n.y.p. |
| **22- O- q11.1/ 1-63** | female/ prenatal | AF | Pat | 47,XX,+mar[100%] | **inv dup(22)(q11.1)** | n.y.p. |
| **22- O- q11.1/ 1-64** | female/ prenatal | AF | Pat | 47,XX,+mar[100%] | **inv dup(22)(q11.1)** | n.y.p. |
| **22- O- q11.1/ 1-65** | male/ prenatal | AF | Mat | 47,XY,+mar[100%] | **inv dup(22)(q11.1)** | n.y.p. |
| **22- O- q11.1/ 1-66** | female/ 37y | PBL | n.a. | 47,XX,+mar[100%] | **inv dup(22)(q11.1)** | n.y.p. |
| **22- O- q11.1/ 1-68** | female/ prenatal | AF | n.a. | 47,XX,+mar[14]/ 46,XX[9] | **inv dup(22)(q11.1)** | n.y.p. |
| **22- O- q11.1/ 1-69** | female/ 47y | PBL | n.a. | 47,XX,+mar[17]/45,X[2]/ 46,X,+mar[1]/46,XX[9] | **inv dup(22)(q11.1)** | n.y.p. |
| **22- O- q11.1/ 1-70** | female/ 37y | PBL | n.a. | 47,XX,+mar[14]/ 46,XX[18] | **inv dup(22)(q11.1)** | n.y.p. |
| **22- O- q11.1/ 1-71** | female/ 17y | PBL | n.a. | 47,XX,+mar[100%] | **inv dup(22)(q11.1)** | n.y.p. |
| **22- O- q11.1/ 1-72** | male/ 50y | PBL | n.a. | 47,XY,+mar[100%] | **inv dup(22)(q11.1)** | n.y.p. |
| **22- O- q11.1/ 1-73** | male/ 44y | PBL | n.a. parental, as brother has same sSMC | 47,XY,+mar[100%] | **inv dup(22)(q11.1)** | n.y.p. |
| **22- O- q11.1/ 1-74** | female/ 40y | PBL | n.a. | 47,XX,+mar[100%] | **inv dup(22)(q11.1)** | n.y.p. |
| **22- O- q11.1/ 1-75** | female/ adult | PBL | n.a. | 47,XX,+mar[100%] | **inv dup(22)(q11.1)** | n.y.p. |

**Table S3a (ctd.)**

| **case no.** | **gender/ age at diagnosis** | **studied material** | **de novo/ inherited** | **GTG-banding result grade of mosaicism** | **final result of the sSMC** | [**Reference**](http://cs-tl.de/DB/CA/sSMC/22/z-Ref.html) |
| --- | --- | --- | --- | --- | --- | --- |
| **22- O- q11.1/ 1-76** | male/ 36y | PBL | n.a. | 47,XY,+mar[100%] | **inv dup(22)(q11.1)** | n.y.p. |
| **22- O- q11.1/ 1-78** | male/ 32y | PBL | n.a. | 47,XY,+mar[100%] | **inv dup(22)(q11.1)** | n.y.p. |
| **22- O-** **q11.1/** **2-1** | female/ prenatal | PBL | de novo | 47,XX,+mar[13]/ 46,XX[27] | **min(22)(:p13**→**q11.1:)** | [{2}](http://cs-tl.de/DB/CA/sSMC/22/z-Ref.html) |
| **22- O-** **q11.1/** **2-2** | female/ prenatal | AF | paternal | 47,XX,+mar[100%] | **min(22)(pter**→**q11.1:)** | n.y.p. |
| **22- O- q11.1/ 2-3** | female/  1y | PBL | de novo | 47,XX,+mar[21]/ 46,XX[29] | **min(22)(:p12**→**q11.1:)** | [{0}](http://cs-tl.de/DB/CA/sSMC/22/z-Ref.html) |
| **22- O- q11.1/ 2-4** | male/ 30y | PBL | n.a. | 47,XY,+mar[100%] | **min(22)(:p12**→**q11.1:)** | n.y.p. |
| **22- O- q11.1/ 2-5** | male/ 37y | PBL | n.a. | 47,XY,+mar[100%] | **min(22)(pter**→**q11.1:)** | n.y.p. |
| **22- O-** **q11.1/** **5-1** | male/ 33y | PBL | n.a. | 47,XY,+mar[100%] | **min(22)(pter**→**q11.1)[5]/ inv dup(22)(q11.1)[5]/ r(22)(::pter**→**q11.1: :q11.1**→**pter::)[2]** | [{212} case 104 {321} case 22-21](http://cs-tl.de/DB/CA/sSMC/22/z-Ref.html) |
| **22- O-** **q11.1/** **5-2** | female/ prenatal | AF | de novo | 47,XX,+mar[100%] | **r(22)(::p1?2**→ **q11.1::)[2]/ min(22)(pter**→**q11.1)[1]/ inv dup(22)(q11.1)[6]** | [{247} case 14](http://cs-tl.de/DB/CA/sSMC/22/z-Ref.html) |
| **22-O-** **q11.1/** **6-1** | male/ 27y | PBL | n.a. | 47,XY,+mar[10%]/ 46,XY[90%] | **r(22)(::p1?2**→**q11.1::)** | [{321} case 22-22](http://cs-tl.de/DB/CA/sSMC/22/z-Ref.html) |
| **22- O-** **q11.1/** **6-2** | male/ prenatal | AF | Pat | 47,XY,+mar[100%] | **r(22)(::p1?3**→**q11.1::)** | n.y.p. |
| **22- O-** **q11.1** **~11.2/** **1-2** | female/ prenatal | AF | de novo? | 47,XX,+mar[18]/ 46,XX[3] | **inv dup(22)(q11.1~11.21) FISH-data:CTA-115F6 at 16.37MB on sSMC** | n.y.p. |
| **22- O-** **q11.21/** **1-1** | female/ prenatal | AF | Mat; familial | 47,XX,+mar[10] | **inv dup(22)(q11.21)** | [{181} case 22-17 {192} case E](http://cs-tl.de/DB/CA/sSMC/22/z-Ref.html) |

**Table S3a (ctd.)**

| **case no.** | **gender/ age at diagnosis** | **studied material** | **de novo/ inherited** | **GTG-banding result grade of mosaicism** | **final result of the sSMC** | [**Reference**](http://cs-tl.de/DB/CA/sSMC/22/z-Ref.html) |
| --- | --- | --- | --- | --- | --- | --- |
| **22- O-** **q11.1** **~11.2/** **1-2** | female/ prenatal | AF | de novo? | 47,XX,+mar[18]/ 46,XX[3] | **inv dup(22)(q11.1~11.21) FISH-data:CTA-115F6 at 16.37MB on sSMC** | n.y.p. |
| **22- O-** **q11.21/** **1-1** | female/ prenatal | AF | Mat; familial | 47,XX,+mar[10] | **inv dup(22)(q11.21)** | [{181} case 22-17 {192} case E](http://cs-tl.de/DB/CA/sSMC/22/z-Ref.html) |
| **22- O-** **q11.21/** **1-3** | female/ adult | PBL | n.a. | 47,XX,+mar[100%] | **inv dup(22)(q11.21) FISH-data:CTA-115F6 at 16.35MB on sSMC** | n.y.p. |
| **22- O-** **q11.21/** **1-6** | male/ 31y | PBL | n.a. | 47,XY,+mar[100%] | **inv dup(22)(q11.21)** | [{321} case 22-24](http://cs-tl.de/DB/CA/sSMC/22/z-Ref.html) |
| **22- O-** **q11.21/** **2-1** | male and female/ children and adult | AF, PBL | familial | 47,+mar[100%] | **inv dup(22)(pter**→ **q11.21: :q11.1**→**pter) FISH-data: CTA-115F6 at 16.35MB on sSMC between 16.35MB and 17.10MB** | [{345} case 11](http://cs-tl.de/DB/CA/sSMC/22/z-Ref.html) |
| **22- O-** **q11.21/** **4-1** | female/ prenatal | AF | Pat | 47,XX,+mar[35]/ 46,XX[12] | **r(22)(::p12**→**q11.21::) FISH-data: RP11-172D7 at 16.37MB on sSMC** | n.y.p. |
| **22- O-** **q11.21/** **4-2** | male/ prenatal | AF | de novo | 47,XY,+mar[46]/ 46,XY[19] | **min(22)(:p11.2**→**q11.21:)** | n.y.p. |
| **22- O-** **q11.21/** **4-3** | male/ 29y | PBL | n.a. | 47,XY,+mar[15]/ 46,XY[15] | **min(22)(pter**→**q11.21:) in aCGH no euchromatin detected** | [{321} case 22-25](http://cs-tl.de/DB/CA/sSMC/22/z-Ref.html) |
| **22- O- q11.21/ 4-5** | female/ adult | PBL | n.a. | 47,XX,+mar[20%]/ 46,XX[80%] | **min(22)(:p11.2→q11.21:) aCGH: arr[hg19]: break in 18,848,020** | n.y.p. |
| **22- O-** **q11.21/** **5-1** | female/ prenatal | AF | maternal (?) (1 sSMC in 50 PBL cells) | 47,XX,+mar[21]/ 46,XX[6] | **r(22)(:p12**→ **q11.21:)[9]/ r(22)(::p12**→ **q11.21::)[3]/ r(22;22)(::p12**→ **q11.21: :p12**→**q11.21::)[3] array: 15.31-16.10 FISH-data: CTA-115F6 at 16.35MB on sSMC** | n.y.p. |
| **22-** **CO- 4** | male/ 1m | PBL | maternal? 46,XX,t(22;?) | 47,XY,+mar[100%] | **inv dup(22)(:q1?2**→**p11.1: :p11.1**→**q1?2:)** | n.y.p. |

**Table S3b**

Small supernumerary marker chromosomes derived from chromosome 22 with associated clinical signs – only not yet published cases from authors’ laboratory.

Case numbers acc. to sSMC database <http://cs-tl.de/DB/CA/sSMC/0-Start.html>
References acc. to <http://cs-tl.de/DB/CA/sSMC/22/z-Ref.html>
Additional case details can be found at <http://cs-tl.de/DB/CA/sSMC/22/c-abnorm.html>

| **case no.** | **gender/ age at diagnosis** | **studied material** | **de novo/ inherited** | **GTG-banding result grade of mosaicism** | **final result of the sSMC** | [**Reference**](http://cs-tl.de/DB/CA/sSMC/22/z-Ref.html) |
| --- | --- | --- | --- | --- | --- | --- |
| **22- W-** **q11.1/** **1-4** | male/ postnatal | PBL | n.a. | 47,XY,+mar[?%] | **inv** **dup(22)(q11.1)** | n.y.p. |
| **22- W-** **q11.1/** **1-5** | female/ 6y | PBL | n.a. | 47,XX,+mar[100%] | **inv** **dup(22)(q11.1)** | n.y.p. |
| **22- W-** **q11.1/** **1-6** | female/ 2y | PBL | n.a. | 47,XX,+mar[100%] | **inv** **dup(22)(q11.1)** | n.y.p. |
| **22- W-** **q11.1/** **3-1** | female/ 7y | PBL | n.a. | 47,XX,+mar[13]/ 46,XX[28] | **r(22)(::p1?3**→**q11.1::)[7]/ inv dup22(q11.1)[5]/ r(22;22)(::p1?3**→**q11.1: :p1?3**→**q11.1)[1]** | n.y.p. |
| **22- W-** **q11.2/** **1-3** | male/ 2m | PBL | de novo | 47,XY,+mar[100%] | **min(22)(pter**→**q11.2:)** | n.y.p. |
| **22- W-** **q11.2/** **1-4** | female/ prenatal | AF | de novo | 47,XX,+mar[100%] | **min(22)(pter**→**q11.2:)** | n.y.p. |
| **22- W-** **q11.21/** **1-1** | male/ postnatal | PBL cell line at ECACC AL0016 | n.a. | 47,XY,+mar[100%] | **min(22)(pter**→**q11.21:)** only first CES-specific BAC (B81B3) present | [{184} case 13](http://cs-tl.de/DB/CA/sSMC/22/z-Ref.html) |
| **22- W-** **q11.21/** **2-1** | female/ postnatal | PBL cell line at ECACC CC0155 | n.a. | 47,XX,+mar[100%] | **dic(22)(pter**→**q11.21: :p11.2**→**q11.21:)** | [{184} case 14](http://cs-tl.de/DB/CA/sSMC/22/z-Ref.html) |
| **22- W-** **q11.21/** **4-1** | female/ 1y | PBL | n.a. | 47,XX,+mar[100%] | **der(22)(pter**→**q11.21: :p11.?2**→**pter)** | n.y.p. |
| **22- W-** **q12.1/** **1-1** | male/ 1y | PBL | de novo | 47,XY,+mar[22]/ 46,XY[6] | **min(22)(pter**→**q12.1:) FISH-data: pter to 24.6MB distal from 20.75Mb** | [{345} case 12](http://cs-tl.de/DB/CA/sSMC/22/z-Ref.html) |

**Table S3b (ctd.)**

| **case no.** | **gender/ age at diagnosis** | **studied material** | **de novo/ inherited** | **GTG-banding result grade of mosaicism** | **final result of the sSMC** | [**Reference**](http://cs-tl.de/DB/CA/sSMC/22/z-Ref.html) | |
| --- | --- | --- | --- | --- | --- | --- | --- |
| **22- W-** **q13.3/** **1-1** | n.a./ postnatal | PBL | n.a. | 47,+r[100%] | **r(22)(::p11.2**→**q13.3::) array: 15.31-49.34 MB** | n.y.p. | |
| **22/8- Wder- 12** | male/ 6y | PBL | n.a. | 47,XY,+mar[100%] | **der(22)t(8;22)(q24.1;q11.2) array-data: 8q24.13-8q24.3 (125,641,226-146,250,824 MB) and 22q11q11.21 (~15,448,000-19,049171 MB)** | [{299}](http://cs-tl.de/DB/CA/sSMC/22/z-Ref.html) | |
| **22- Wder- 1** | Emanuel syndrome  prenatal | | | | | | [{1} case 29; {3} case shown in Fig. 5](http://cs-tl.de/DB/CA/sSMC/22/z-Ref.html) |
| **22- Wder- 143** | Emanuel syndrome  postnatal | | | | | | n.y.p. |
| **22- Wder- 236** | Emanuel syndrome  postnatal | | | | | | [{181} case 5](http://cs-tl.de/DB/CA/sSMC/22/z-Ref.html) |
| **22- Wder- 241** | Emanuel syndrome  postnatal | | | | | | n.y.p. |
| **22- Wder- 243** | Emanuel syndrome  prenatal | | | | | | n.y.p. |
| **22- Wder- 244** | Emanuel syndrome  postnatal | | | | | | [{216} cases 38](http://cs-tl.de/DB/CA/sSMC/22/z-Ref.html) |
| **22- Wder- 245** | Emanuel syndrome  postnatal | | | | | | [{216} cases 39](http://cs-tl.de/DB/CA/sSMC/22/z-Ref.html) |
| **22- Wder- 246** | Emanuel syndrome  postnatal | | | | | | [{216} cases 40](http://cs-tl.de/DB/CA/sSMC/22/z-Ref.html) |
| **22- Wder- 250** | Emanuel syndrome  prenatal | | | | | | [{247} case 16](http://cs-tl.de/DB/CA/sSMC/22/z-Ref.html) |
| **22- Wder- 251** | Emanuel syndrome  prenatal | | | | | | n.y.p. |
| **22- Wder- 326** | Emanuel syndrome  prenatal | | | | | | n.y.p. |
| **22- Wder- 338** | Emanuel syndrome  prenatal | | | | | | n.y.p. |
| **22- Wder- 385** | Emanuel syndrome  prenatal | | | | | | n.y.p. |

**Table S3b (ctd.)**

| **case no.** | **gender/ age at diagnosis** | **studied material** | **de novo/ inherited** | **GTG-banding result grade of mosaicism** | **final result of the sSMC** | [**Reference**](http://cs-tl.de/DB/CA/sSMC/22/z-Ref.html) | |
| --- | --- | --- | --- | --- | --- | --- | --- |
| **22- Wder- 386** | Emanuel syndrome  postnatal | | | | | | n.y.p. |
| **22- Wder- 393** | Emanuel syndrome  prenatal | | | | | | n.y.p. |
| **22-** **Wces-** **1** | Cat eye syndrome  postnatal | | | | | | [{1} case 30](http://cs-tl.de/DB/CA/sSMC/22/z-Ref.html) |
| **22- Wces- 5-74** | Cat eye syndrome  prenatal | | | | | | n.y.p. |
| **22- Wces- 5-85** | Cat eye syndrome  postnatal | | | | | | n.y.p. |
| **22- Wces- 5-86** | Cat eye syndrome  prenatal | | | | | | [{275} case 23](http://cs-tl.de/DB/CA/sSMC/22/z-Ref.html) |
| **22- Wces- 5-88** | Cat eye syndrome  prenatal | | | | | | n.y.p. |
| **22- Wces- 5-90** | Cat eye syndrome  prenatal | | | | | | n.y.p. |
| **22- Wces- 5-91** | Cat eye syndrome  prenatal | | | | | | n.y.p. |
| **22- Wces- 5-93** | Cat eye syndrome  postnatal | | | | | | [{197} case 14; {345} case 10](http://cs-tl.de/DB/CA/sSMC/22/z-Ref.html) |
| **22- Wces- 5-94** | Cat eye syndrome  postnatal | | | | | | [{275} case 24 {283} case Si-5](http://cs-tl.de/DB/CA/sSMC/22/z-Ref.html) |
| **22- Wces- 5-95** | Cat eye syndrome  # **aCGH: 0.00-17.00 MB FISH-data incl 16.2 MB**  postnatal | | | | | | [{283} case Si-6](http://cs-tl.de/DB/CA/sSMC/22/z-Ref.html) |
| **22- Wces- 5-96** | Cat eye syndrome  postnatal | | | | | | n.y.p. |
| **22- Wces- 5-97** | Cat eye syndrome  postnatal | | | | | | n.y.p. |
| **22- Wces- 5-98** | Cat eye syndrome  prenatal | | | | | | n.y.p. |

**Table S3b (ctd.)**

| **case no.** | **gender/ age at diagnosis** | **studied material** | **de novo/ inherited** | **GTG-banding result grade of mosaicism** | **final result of the sSMC** | [**Reference**](http://cs-tl.de/DB/CA/sSMC/22/z-Ref.html) | |
| --- | --- | --- | --- | --- | --- | --- | --- |
| **22- Wces- 5-100** | Cat eye syndrome  prenatal | | | | | | n.y.p. |
| **22- Wces- 5-101** | Cat eye syndrome **+dic(13/21;22)(13/21pter→13/21q11: :22q11.1~11.2**→**22q11.21~11.22: :22q11.21~11.22**→**22pter)**  postnatal | | | | | | [{213} case 10 {299}](http://cs-tl.de/DB/CA/sSMC/22/z-Ref.html) |
| **22- Wces- 5-106** | Cat eye syndrome  postnatal | | | | | | n.y.p. |
| **22- Wces- 5-107** | Cat eye syndrome  postnatal | | | | | | n.y.p. |
| **22- Wces- 5-109** | Cat eye syndrome **aCGH: 0.00-17.00 MB aCGH midi: 0-17,274,894 MB**  postnatal | | | | | | n.y.p. |
| **22- Wces- 5-112** | Cat eye syndrome  prenatal | | | | | | n.y.p. |
| **22- Wces- 5-114** | Cat eye syndrome  prenatal | | | | | | n.y.p. |
| **22- Wces- 5-117** | Cat eye syndrome  prenatal | | | | | | n.y.p. |
| **22- Wces- 5-118** | Cat eye syndrome  prenatal | | | | | | n.y.p. |
| **22- Wces- 5-120** | Cat eye syndrome  postnatal | | | | | | n.y.p. |
| **22- Wces- 5-121** | Cat eye syndrome  prenatal | | | | | | n.y.p. |
| **22- Wces- 5-126** | Cat eye syndrome  postnatal | | | | | | n.y.p. |
| **22- Wces- 5-127** | Cat eye syndrome **aCGH: 0.00-19.78 MB (hg18, NCBI Build 36)**  postnatal | | | | | | n.y.p. |
| **22- Wces- 5-130** | Cat eye syndrome  prenatal | | | | | | [{275} case 25](http://cs-tl.de/DB/CA/sSMC/22/z-Ref.html) |
| **22- Wces- 5-131** | Cat eye syndrome  prenatal | | | | | | n.y.p. |

**Table S3b (ctd.)**

| **case no.** | **gender/ age at diagnosis** | **studied material** | **de novo/ inherited** | **GTG-banding result grade of mosaicism** | **final result of the sSMC** | [**Reference**](http://cs-tl.de/DB/CA/sSMC/22/z-Ref.html) | |
| --- | --- | --- | --- | --- | --- | --- | --- |
| **22- Wces- 5-145** | Cat eye syndrome  prenatal | | | | | | n.y.p. |
| **22- Wces- 5-146** | Cat eye syndrome  prenatal | | | | | | n.y.p. |
| **22- Wces- 5-147** | Cat eye syndrome  postnatal | | | | | | n.y.p. |
| **22- Wces- 5-159** | Cat eye syndrome  postnatal | | | | | | n.y.p. |
| **22- Wces- 5-160** | Cat eye syndrome  **array-data: pter to 17.19 MB**  postnatal | | | | | | n.y.p. |
| **22- Wces- 5-162** | Cat eye syndrome  **array-data: pter to 17.19 MB**  postnatal | | | | | | n.y.p. |
| **22- Wces- 5-164** | Cat eye syndrome  prenatal | | | | | | n.y.p.. |
| **22- Wces- 5-165** | Cat eye syndrome  prenatal | | | | | | n.y.p. |
| **22- Wces- 5-166** | Cat eye syndrome  prenatal | | | | | | n.y.p. |
| **22- Wces- 5-167** | Cat eye syndrome **array-data: pter to 17.02 MB**  prenatal | | | | | | n.y.p. |
| **22- Wces- 5-170** | Cat eye syndrome  prenatal | | | | | | n.y.p. |
| **22- Wces- 5-175** | Cat eye syndrome  postnatal | | | | | | n.y.p. |
| **22- Wces- 5-178** | Cat eye syndrome  prenatal | | | | | | n.y.p. |
| **22- Wces- 5-187** | Cat eye syndrome  prenatal | | | | | | n.y.p. |
| **22- Wces- 5-192** | Cat eye syndrome  postnatal | | | | | | [{358}](http://cs-tl.de/DB/CA/sSMC/22/z-Ref.html) |

**Table S3b (ctd.)**

| **case no.** | **gender/ age at diagnosis** | **studied material** | **de novo/ inherited** | **GTG-banding result grade of mosaicism** | **final result of the sSMC** | [**Reference**](http://cs-tl.de/DB/CA/sSMC/22/z-Ref.html) | |
| --- | --- | --- | --- | --- | --- | --- | --- |
| **22- Wces- 5-196** | Cat eye syndrome  postnatal | | | | | | n.y.p. |
| **22- Wces- 5-197** | Cat eye syndrome  postnatal | | | | | | n.y.p. |
| **22- Wces- 5-200** | Cat eye syndrome 47,XX,+der(22)(:q12.?1→p11.2: :p11.2→q12.?1:)[20%]/46,XX[80%] postnatal | | | | | | [{358}](http://cs-tl.de/DB/CA/sSMC/22/z-Ref.html) |
| **22- Wces- 5-202** | Cat eye syndrome  postnatal | | | | | | n.y.p. |
| **22- Wces- 5-203** | Cat eye syndrome  postnatal | | | | | | n.y.p. |
| **22- Wces- 5-207** | Cat eye syndrome  prenatal | | | | | | n.y.p. |
| **22- Wces- 5-208** | Cat eye syndrome  postnatal | | | | | | n.y.p. |
| **22- Wces- 5-210** | Cat eye syndrome  postnatal | | | | | | n.y.p. |
| **22- Wces- 5-211** | Cat eye syndrome  postnatal | | | | | | n.y.p. |
| **22- Wces- 7-1** | Cat eye syndrome  postnatal | | | | | | n.y.p. |
| **22- Wces- 7-2** | Cat eye syndrome normal, but daughter with suspicion of syndrome  postnatal | | | | | | n.y.p. |
| **22- Uc- 14 (7)** | female/ prenatal | PBL | n.a. | 47,XX,+mar[100%] | **der(22)t(7;22)(p15.3;q11.2)** | n.y.p. | |
| **22- Uc- 15 (7)** | male/ prenatal | AF | n.a. | 47,XY,+mar[100%] | **der(22)t(7;22)(p11.2;q11.1)** | n.y.p. | |
| **22- Ud- 2** | male/ prenatal | AF | de novo | 47,XY,+mar[100%] | **der(22)(pter→q11.21: :q11.23→q1?21: :q11.1→pter)** | n.y.p. | |

**Table S3c**

Small supernumerary marker chromosomes derived from chromosome 22 without clear clinical information and/or correlation – only not yet published cases from authors’ laboratory.

Case numbers acc. to sSMC database <http://cs-tl.de/DB/CA/sSMC/0-Start.html>
References acc. to <http://cs-tl.de/DB/CA/sSMC/22/z-Ref.html>
Additional case details can be found at <http://cs-tl.de/DB/CA/sSMC/22/d-uncl.html>

| **case no.** | **gender/ age at diagnosis** | **studied material** | **de novo/ inherited** | **GTG-banding result grade of mosaicism** | **final result of the sSMC** | [**Reference**](http://cs-tl.de/DB/CA/sSMC/22/z-Ref.html) |
| --- | --- | --- | --- | --- | --- | --- |
| **22-** **U- 1** | female/ prenatal | AF | de novo | 47,XX,+mar[20] | **inv dup(22)(q11.1)** | [{4} case 12](http://cs-tl.de/DB/CA/sSMC/22/z-Ref.html) |
| **22-** **U- 2** | female/ prenatal | AF | paternal; (mar in 2/50 mitosis) | 47,XX,+mar[6]/ 46,XX[35] | **inv**.**dup(22)(q11.2) [cep22+,cep14/22+, Midi54+,bK115F6+]/ der(22)(:p11.1**→**q11.1:) [cep22+,cep14/22-]/ der(22)(:p11.1q11.1:) [cep22+,cep14/22+]** | [{1} case 33](http://cs-tl.de/DB/CA/sSMC/22/z-Ref.html) |
| **22-** **U- 10** | female/ prenatal | AF | de novo | 47,XX,+mar[~15%] | **min(22)(:p11.1**→**q11.1).ish (D22Z4+;D14/22Z1-)** | [{175}](http://cs-tl.de/DB/CA/sSMC/22/z-Ref.html) |
| **22-** **U- 15** | n.a./ prenatal | AF | paternal (?) 5/150 with mar | 47,+mar[40%]/ 46[60%] | **inv dup(22)(q10)** | n.y.p. |
| **22-** **U- 16** | female/ prenatal | AF | n.a. | 47,XX,+mar[25%]/ 46,XX[75%] | **inv dup(22)(q10)** | n.y.p. |
| **22-** **U- 35** | female/ prenatal | AF | n.a. | 47,XX,der(22q),+mar)/ 46,XX,der(22q) | **min(22)(p11.1q11.1)** | [{216} case 35](http://cs-tl.de/DB/CA/sSMC/22/z-Ref.html) |
| **22-** **U- 38** | female/ prenatal | AF | n.a. | 47,XX,+mar[50%]/ 46,XX[50%] | **min(22)(:p11.1**→**q11.23:)** | n.y.p. |
| **22-** **U- 39** | male/ prenatal | AF | n.a. | 47,XY,+mar[100%] | **inv dup(22)(q11.1)** | n.y.p. |
| **22-** **U- 41** | male/ 1y | PBL | mat mother has SMC up to 3 times | 47,XY,+mar[100%] | **inv dup(22)(q11.1)** | n.y.p. |
| **22-** **U- 44** | female/ prenatal | AF | n.a. | 47,XX,+mar[100%] | **inv dup(22)(q11.1)** | n.y.p. |
| **22-** **U- 45** | male/ prenatal | AF | n.a. | 47,XY,+mar[35%]/ 46,XY[65%] | **inv dup(22)(q11.1)** | n.y.p. |
| **22-** **U- 46** | male/ prenatal | AF | n.a. | 47,XY,+mar[100%] | **inv dup(22)(q11.1)** | n.y.p. |
| **22-** **U- 46a** | female/ prenatal | AF | n.a. | 47,XX+mar[59]/ 46,XX[41] | **inv dup(22)(q11.1)** | n.y.p. |

**Table S3c (ctd.)**

| **case no.** | **gender/ age at diagnosis** | **studied material** | **de novo/ inherited** | **GTG-banding result grade of mosaicism** | **final result of the sSMC** | [**Reference**](http://cs-tl.de/DB/CA/sSMC/22/z-Ref.html) |
| --- | --- | --- | --- | --- | --- | --- |
| **22-** **U- 46b** | female/ prenatal | AF | n.a. | 47,XX,+mar[100%] | **inv dup(22)(q11.1)** | n.y.p. |
| **22-** **U- 47** | female/ prenatal | AF | n.a. | 47,XX,+mar[95%]/ 46,XX[5%] | **min(:p1?3**→**q11.1:)[6]/ r(22)(::p1?3**→**q11.1::)[4]** | n.y.p. |
| **22-** **U- 48** | n.a./ prenatal | AF | n.a. | 47,+mar[?%]/ 46[?%] | **der(22)(:q11.1**→**p11.2: :p11.2**→**q11.1:)** | n.y.p. |
| **22-** **U- 49** | n.a./ prenatal | AF | n.a. | 47,+mar[?%]/ 46[?%] | **min(22)(pter**→**q11.21:)** | n.y.p. |
| **22- U- 55** | male/ prenatal | AF | n.a. | 47,XY,+mar[100%] | **inv dup(22)(q11.1)** | [{282} case 3](http://cs-tl.de/DB/CA/sSMC/22/z-Ref.html) |
| **22- U- 56** | female/ prenatal | AF | de novo | 47,XX,+mar[100%] | **min(22)(pter**→**q13.?1:)** | n.y.p. |
| **22- U- 62** | male/ prenatal | AF | n.a. | 47,XY,+mar[80%]/ 46,XY[20%] | **inv dup(22)(q11.1)** | n.y.p. |
| **22- U- 63** | male/ prenatal | AF | n.a. | 47,XY,+mar[100%] | **inv dup(22)(q11.1)** | n.y.p. |
| **22- U- 71** | male/ prenatal | AF | de novo | 47,XY,+mar[100%] | **inv dup(22)(q11.1)** | n.y.p. |
| **22- U- 72** | female/ prenatal | AF | n.a. | 47,XX,+mar[100%] | **inv dup(22)(q11.1)** | n.y.p. |
| **22- U- 73** | female/ prenatal | AF | n.a. | 47,XX,+mar[50%]/ 46,XX[50%] | **min(22)(:p11.2→q11.1:)** | n.y.p. |
| **22- U- 74** | male/ prenatal | CH AF | n.a. | 47,XY,+mar[24]/ 46,XY[7] in AF 100% | **inv dup(22)(q11.1)** | n.y.p. |
| **22- U- 75** | female/ prenatal | AF | n.a. | 47,XX,+mar[6]/ 46,XX[15] | **inv dup(22)(q11.1)** | n.y.p. |
| **22- U- 76** | male/ prenatal | AF | n.a. | 47,XY,+mar[100%] | **inv dup(22)(q11.1)** | n.y.p. |
| **22- U- 77** | male/ prenatal | AF | n.a. | 47,XY,+mar[4]/ 46,XY[16] | **der(22)(:p11.2→q11.1: :q11.1→p11.2:)** | n.y.p. |
| **22- U- 78** | male/ prenatal | CH | n.a. | 47,XY,+mar[46%]/ 46,XY[54%] | **der(22)(:p11.2→q10:)** | n.y.p. |
| **22- U- 79** | male/ prenatal | AF | n.a. | 47,XY,+mar[7]/ 46,XY[9] | **inv dup(22)(q11.1)** | n.y.p. |

**Table S3c (ctd.)**

| **case no.** | **gender/ age at diagnosis** | **studied material** | **de novo/ inherited** | **GTG-banding result grade of mosaicism** | **final result of the sSMC** | [**Reference**](http://cs-tl.de/DB/CA/sSMC/22/z-Ref.html) |
| --- | --- | --- | --- | --- | --- | --- |
| **22- U- 80** | male/ postnatal | PBL | n.a. | 48,XY,+marx2[1]/ 47,XY,+mar[5]/46,XY[1] | **inv dup(22)(q11.1)** | n.y.p. |
| **22- U- 85** | female/ prenatal | Ch | de novo | 47,XX,+mar[22]/ 46,XX[10] | **inv dup(22)(q11.1)** | n.y.p. |
| **22- U- 86** | male/ prenatal | Ch | n.a. | 47,XY,+mar[~50%]/ 46,XY[~50%] | **min(22)(:p11.1**→**q11.21:)** | n.y.p. |
| **22- U- 87** | male/ prenatal | AF | n.a. | 48,XY,+mar,+mar[?%]/ 47,XY,+mar[?%]/46,XY[?%] | **inv dup(22)(q11.1)** | n.y.p. |
